# Depleting receptor tyrosine kinases EGFR and HER2 overcomes resistance to EGFR inhibitors in colorectal cancer

**DOI:** 10.1186/s13046-022-02389-z

**Published:** 2022-06-02

**Authors:** Lu Yang, Arup Bhattacharya, Yun Li, Sandra Sexton, Xiang Ling, Fengzhi Li, Yuesheng Zhang

**Affiliations:** 1 Department of Pharmacology and Therapeutics, Roswell Park Comprehensive Cancer Center, Buffalo, NY 14263 USA; 2 Department of Pharmacology and Toxicology, and Massey Cancer Center, Virginia Commonwealth University School of Medicine, Richmond, VA 23298 USA; 3 Department of Urology, Roswell Park Comprehensive Cancer Center, Buffalo, NY 14263 USA; 4 Department of Animal Resources, Roswell Park Comprehensive Cancer Center, Buffalo, NY 14263 USA

**Keywords:** Aderbasib, Cetuximab, Colorectal cancer, EGFR, HER2, Panitumumab, PEPD^G278D^, Therapeutic resistance

## Abstract

**Background:**

Epidermal growth factor receptor (EGFR) inhibitors, including cetuximab and panitumumab, are valuable therapeutics for colorectal cancer (CRC), but resistance to these inhibitors is common. The reason for such resistance is not well understood, which hampers development of better therapeutic strategies. Although activating mutations in KRAS, BRAF and PIK3CA are considered major drivers of CRC resistance to EGFR inhibitors, therapeutic targeting of these drug resistance drivers has not produced substantial clinical benefit.

**Methods:**

We exploited cell lines and mouse tumor models (cell line xenografts and patient derived xenografts) for experiments of genetic and pharmacologic depletion of EGFR and/or its family member HER2, including EGFR mutants, inhibition of EGFR ligand shedding, and biochemical analysis of signaling proteins, to delineate the mechanism of CRC resistance to EGFR inhibitors and to assess the therapeutic activity of PEPD^G278D^, which is a recombinant human protein that induces the degradation of both EGFR and HER2.

**Results:**

The sensitivity of CRC cells to cetuximab and panitumumab correlates with the ability of these drugs to induce EGFR downregulation. PEPD^G278D^ strongly inhibits oncogenic signaling and growth of CRC cells by causing profound depletion of EGFR and HER2, regardless of activating mutations of KRAS, BRAF and PIK3CA. siRNA knockdown of EGFR or HER2 also inhibits CRC cells resistant to EGFR inhibitors. Tumors harboring mutated KRAS, BRAF and/or PIK3CA also overexpress EGFR ligands, further suggesting that EGFR signaling remains important to the tumors. While excessive tumor-generated high-affinity EGFR ligands block target engagement by PEPD^G278D^, aderbasib, an inhibitor of ADAM10 and ADAM17, enables PEPD^G278D^ to exert strong antitumor activity by inhibiting ligand shedding. Moreover, adding fluorouracil, which is commonly used in CRC treatment, to the combination of PEPD^G278D^ and aderbasib further enhances tumor inhibition.

**Conclusions:**

Our study shows that CRC resistance to EGFR inhibitors results primarily from the inability of the inhibitors to downregulate their target and that a PEPD^G278D^-based combination treatment overcomes the resistance.

**Supplementary Information:**

The online version contains supplementary material available at 10.1186/s13046-022-02389-z.

## Background

Epidermal growth factor receptor (EGFR), a receptor tyrosine kinase (RTK), is a well-known oncogenic driver and a therapeutic target in several types of human cancer. It functions by forming homodimeric and heterodimeric signaling units, which activate various oncogenic signaling pathways in cancer cells. More than 80% of primary and metastatic colorectal cancers (CRCs) are EGFR-positive, with overexpression in about 60% and gene amplification in about 10% of the cases [1, 2]. Two classes of EGFR inhibitors are available clinically, including tyrosine kinase inhibitors (TKIs) and monoclonal antibodies (MABs). However, EGFR TKIs have not shown significant therapeutic activity in CRC [3]. Two EGFR-directed MABs are approved for treatment of patients with CRC, including cetuximab and panitumumab, but only 10–20% of the patients respond to treatment and response lasts typically 3–12 months [4, 5]. The two MABs show similar efficacy in CRC patients [6]. The molecular basis underlying response to the MABs is not well known. Many mechanisms that confer primary or acquired resistance to the EGFR inhibitors in CRC have been reported, including but not limited to activating mutations of KRAS, NRAS, BRAF, and PIK3CA [7, 8]. Most notably, activating KRAS mutations occur in up to nearly half of metastatic CRC cases [9, 10], and this group of patients do not benefit from the EGFR MABs [11, 12]. It is widely believed that CRC cells are rendered independent of EGFR by changes in other signaling molecules. However, response to therapeutic targeting of KRAS, BRAF or PIK3CA is very limited if any [13–18]. Identifying the key vulnerability of drug-resistant CRC cells and developing therapeutic strategies targeting such vulnerability remain critically important.

In the present study, we compared the response of a panel of CRC cell lines to cetuximab, panitumumab, and PEPD^G278D^. PEPD^G278D^ is a recombinant and enzymatically inactive mutant of human peptidase D (PEPD), with replacement of glycine 278 by aspartic acid. We recently showed that PEPD^G278D^ induces the internalization and degradation of both EGFR and its family member HER2 by binding directly to their extracellular domains [19–21]. We also showed that PEPD^G278D^ inactivates other RTKS indirectly by disrupting their heterodimerization with EGFR or HER2 [21, 22]. Consequently, PEPD^G278D^ strongly inhibits the growth of cancer cells and tumors overexpressing EGFR and/or HER2 [19–22]. EGFR and HER2 appear to be the only direct targets of PEPD^G278D^, as we showed that cells and tumors lacking these RTKs do not respond to PEPD^G278D^ [19–22]. The CRC cell lines used in this study express EGFR and HER2 at different levels or have no expression of these RTKs. Some of the cell lines harbor activating mutations of KRAS, BRAF and/or PIK3CA. siRNA knockdown of EGFR or HER2 was carried out to assist data interpretation. We also evaluated the effect of PEPD^G278D^ on EGFR mutants which occur in patient CRC and are insensitive to cetuximab or panitumumab. Moreover, we compared the inhibitory activities of cetuximab and PEPD^G278D^ in vivo using mouse CRC models with and without mutations of KRAS, BRAF and/or PIK3CA.

Our results show unexpectedly that CRC resistance to cetuximab and panitumumab results primarily from their inability to downregulate EGFR. Presence of activating mutations of KRAS, BRAF and/or PIK3CA does not curtail inhibition of oncogenic signaling and cell growth induced by PEPD^G278D^ via depletion of EGFR and HER2. siRNA knockdown of EGFR or HER2 also inhibits the growth of CRC cells resistant to the EGFR MABs. PEPD^G278D^ targets wild type (WT) and mutated EGFR with similar efficacy. In CRC tumor models resistant to the EGFR MABs, tumor-generated high affinity EGFR ligands compete with PEPD^G278D^ for EGFR binding, but aderbasib, an inhibitor of a disintegrin and metalloproteinase domain-containing protein 10 (ADAM10) and ADAM17 [23], inhibits EGFR ligand shedding from tumor cells and allows target engagement by PEPD^G278D^, resulting in significant inhibition of tumor growth, while aderbasib as a monotherapy is inactive. ADAM10 and ADAM17 are responsible for shedding of all EGFR ligands from cells [24, 25]. Moreover, the antitumor activity of PEPD^G278D^ is further enhanced by fluorouracil (5-FU), which is widely used in CRC treatment. Collectively, our results show that CRC resistance to EGFR inhibitors stems primarily from the inability of the inhibitors to downregulate EGFR, rather than mutations in EGFR, KRAS, BRAF and PIK3CA, and suggest that a therapeutic strategy centered on PEPD^G278D^ is highly promising for overcoming drug resistance in CRC.

## Materials and methods

### Antibodies

The following antibodies were purchased from Cell Signaling: anti-EGFR (Cat # 2232), anti-pY1173-EGFR (Cat # 4407), anti-HER2 (Cat# 2165), anti-pY1221/1222-HER2 (Cat # 2243), anti-HER3 (Cat # 12708), anti-pY1328-HER3 (Cat # 14525), anti-insulin-like growth factor 1 receptor (IGFIR, Cat # 9750), anti-pY1131-IGFIR (Cat # 3021), anti-MET (Cat # 8198), anti-pY1234/1235-MET (Cat # 3077), anti-MEK (Cat # 4694), anti-pS217/221-MEK (Cat # 9154), anti-lysosomal-associated membrane protein 1 (LAMP1, Cat # 15665), anti-AKT (Cat # 4691), anti-pS473-AKT (Cat # 4060), anti-ERK (Cat # 9102), anti-pT202/Y204-ERK (Cat # 9101), anti-SRC (Cat # 2123), anti-pY416-SRC (Cat # 6943), anti-cleaved caspase 3 (Cat # 9661). Anti-His tag (Cat # MA1–21315), goat-anti-mouse secondary antibody conjugated to Alexa-Fluor 488 (Cat # A-11029), and goat anti-rabbit secondary antibody conjugated to Alexa-Fluor 647 (Cat # A32733) were purchased from Thermo Fisher Scientific. Anti-PEPD (Cat # sc-390,042) and anti-NRAS (Cat # sc-31) were purchased from Santa Cruz Biotechnology. Anti-heparin-binding EGF-like growth factor (HB-EGF, Cat# AF-259) and anti-amphiregulin (AREG, Cat # AF-262) were purchased from R&D Systems. Anti-mouse IgG conjugated to horseradish peroxidase (IgG-HRP; Cat # NA931V) and anti-rabbit IgG-HRP (Cat # NA934V) were purchased from GE Healthcare. Anti-PEPD (Cat # ab86507) and anti-KRAS (Cat # WH0003845M1) were purchased from Abcam and Sigma-Aldrich, respectively. Anti-HRAS (Cat # 18295–1-AP) and anti-GAPDH (Cat# MAB374) were purchased from Proteintech and Millipore, respectively.

### Chemicals, biochemicals, and enzymes

Recombinant PEPD^G278D^ was generated in our own lab as previously reported [26]. Briefly, PEPD^G278D^ was synthesized in *E coli* using pBAD/TOPO-PEPD^G278D^-His, purified by nickel chromatography, and concentrated in phosphate-buffered saline (PBS) using Ultracel YM-30 Centricon which was purchased from Millipore (MRCF0R030). Recombinant human HB-EGF (Cat #: 259-HE) was obtained from R&D Systems. Aderbasib was purchased from Medical Isotopes (Cat# 17322). The following chemicals were purchased from Sigma-Aldrich: enoxaparin (EP, Cat# 1235820), 5-FU (Cat# F6627), BSA (Cat# 9048-46-8), paraformaldehyde (Cat# F1635), dimethyl sulfoxide (DMSO; Cat# M81802), chloroquine (Cat# C6628), phenylmethanesulfonyl fluoride (PMSF; 329–98-6), phosphatase inhibitor cocktail 2 (Cat# P5726), phosphatase inhibitor cocktail 3 (Cat# P0044), and methylthiazolyldiphenyl-tetrazolium bromide (MTT, Cat# M2128). Lipofectamine RNAiMAX (Cat# 13778075), lipofectamine 3000 (Cat# L3000–008), and ProLong Gold antifade reagent with DAPI (Cat# P36941) were purchased from Thermo Fisher Scientific. A protease inhibitor cocktail (Cat# 11–836–153-001) was purchased from Roche Applied Science. D-luciferin was purchased from Gold Biotechnology (Cat# LUCK-1G). G-sepharose beads (Cat # 17–6002-35), and Matrigel (Cat# 356237) were purchased from GE Healthcare and Corning, respectively. Sodium dodecyl sulfate (SDS) was purchased from Bio-Rad (Cat# 161–0301). Cell lysis buffer (10x) was purchased from Cell Signaling (Cat# 9803).

### Assay kits

Human Amphiregulin / AREG ELISA Kit PicoKine (Cat# EK0304) and human HB-EGF ELISA Kit (Cat# EK0770) were purchased from Boster Biological. PI3-Kinase Activity ELISA: Pico Kit was purchased from Echelon Biosciences (Cat# K-1000S). Ras Activation ELISA Kit was purchased from Cell Biolabs (Cat# STA-440). BCA Protein Assay Kit (reagent A: Cat# 23228; reagent B: Cat# 1859078) was purchased from Pierce. RNeasy Mini Kit (Cat# 74104), and MinElute PCR Purification Kit (Cat# 28004) were purchased from Qiagen. Luminata Classico (Cat# WBLUC0500), and Luminata Cresendo (Cat# WBLUR0100) were purchased from Millipore.

### Plasmids

pGL4.51[*luc2*/CMV/Neo] was purchased from Promega (Cat# E132A). pCMV6-A-EGFR-puro, reported previously [20], was used as a template to generate single point mutations of EGFR, including R451C, K467T, and S492R, using the QuikChange Lightning Site-Directed Mutagenesis Kit (Agilent Technologies). The primers were purchased from IDT, including primers for generating EGFR^R451C^ (forward: 5′-tgaacataacatccttgggattatgctccctcaagg-3′; reverse: 5′-ccttgagggagcataatcccaaggatgttatgttca-3′), EGFR^K467T^ (forward: 5-ggagatgtgataatttcaggaaacacaaatttgtgctatgcaaatacaata-3′; reverse: 5′-tattgtatttgcatagcacaaatttgtgtttcctgaaattatcacatctcc-3′), and EGFR^S492R^ (forward: 5′-ggtcagaaaaccaaaattataaggaacagaggtgaaaacagc-3′; reverse: 5′-gctgttttcacctctgttccttataattttggttttctgacc-3′). All constructs were confirmed by DNA sequence analysis.

### Genotyping of cell lines and PDX

It was previously shown that HCT116 cells carry KRAS (G13D) and PIK3CA (H1047R) mutations and that HT29 cells carry BRAF (V600E) and PIK3CA (P449T) mutations [27]. Colon PDX14650 was found previously to carry KRAS (G12D) mutation. We carried out experiments to confirm the genetic changes, using SW48 cells and SW620 as negative controls. Total RNA was isolated from each cell line and the PDX using a RNeasy Mini Kit, following manufacturer’s instruction. RNA (500 ng per sample) was reverse transcribed into complementary DNA using a previously described method [21]. The corresponding gene amplicons encompassing KRAS G12 and G13, PIK3CA P449 and H1047, and BRAF V600 were amplified by PCR. The PCR conditions used for all reactions are as follows: 95 °C for 2 min, 35 cycles at 95 °C for 30 sec (denaturation), 64 °C (KRAS G12 and G13, PIK3CA P449) or 54 °C (PIK3CA H1047 and BRAF V600) for 30 sec (annealing), and 72 °C for 30 sec (extension), with the final extension performed at 72 °C for 5 min. The primers were purchased from IDT, including primers for KRAS G12 and G13 (forward: 5′-ccatttcggactgggagcgag-3′; reverse: 5′-gcactgtactcctcttgacctgc-3′), primers for BRAF V600 (forward: 5′-gcacctacacctcagcagtt-3′; reverse: 5′-gacttctggtgccatccaca-3′), primers for PIK3CA P449 (forward: 5′-cccaggtggaatgaatggct-3′; reverse: 5′-accacactgctgaaccagtc-3′), and primers for PIK3CA H1047 (forward: 5′-acagcatgccaatctcttca-3′; reverse: 5′-ttgctgtaaattctaatgctgttc-3′). All PCR reaction products were purified using the MinElute PCR purification Kit, following manufacturer’s instruction and were subjected to DNA sequencing. Each of the forward primers except for PIK3CA H1047 was used for the sequencing analyses across the specific amino acid sites on each target gene. The following primer was used for sequencing across PIK3CA H1047: 5′-aatgatgcttggctctgga-3′.

### Cell culture

HCT116 cells (Cat# CCL-247), HT29 cells (Cat# HTB-38), SW48 cells (Cat# CCL-231), and SW620 cells (Cat# CCL-227) were from American Type Culture Collection. HCT116 cells stably expressing firefly luciferase were generated by transfecting HCT116 cells with pGL4.51[*luc2*/CMV/Neo] and selection under neomycin. HCT116 cells and luciferase-tagged HCT16 cells were cultured in high-glucose Dulbecco’s Modified Eagle Medium (DMEM) supplemented with 10% fetal bovine serum (FBS). HT29 cells were cultured in McCoy’s 5A Medium supplemented with 10% FBS. SW48 cells were cultured in RPMI-1640 medium supplemented with 1% HEPES, 1% Sodium Pyruvate and 10% FBS. SW620 cells were cultured in L-15 medium supplemented with 10% FBS. All cell lines were mycoplasma-free and were authenticated using short tandem repeat. SW620 cells were cultured in humidified incubators at 37 °C with 100% air. All other cells were cultured in humidified incubators at 37 °C with 5% CO_2_. High glucose DMEM (Cat# 10–013-CV), McCoy’s 5A medium (Cat# 10–050-CV), and RPMI-1640 medium (Cat# 10–040-CV) were purchased from Corning Cellgro. L-15 medium was purchased from Thermo Fisher (Cat# 11415064). FBS was purchased from Gibco (Cat# 10437).

### Gene and siRNA transfection, and other treatments

Transfection of pCMV6-A-puro-EGFR, pCMV6-A-puro-EGFR^R451C^, pCMV6-A-puro-EGFR^K467T^ or pCMV6-A-puro-EGFR^RS492R^ was performed using Lipofectamine 3000. SW620 cells were grown in 6-well plates (3 × 10^5^ cells/well with 2 ml medium) for 24 h and then transfected with a plasmid at 1 μg DNA per well for 48 h. For siRNA transfection, cells were grown in 96-well plates (2 × 10^3^ HCT116 cells/well or 6 × 10^3^ HT29 cells/well with 100 μl medium) or 24-well plates (2 × 10^4^ HCT116 cells/well or 6 × 10^4^ HT29 cells/well with 500 μl medium) for 24 h and then transfected with nonspecific scramble siRNA, EGFR siRNA or HER2 siRNA (25 nM) using Lipofectamine RNAiMAX for 48 h. All siRNAs were purchased from Origene. The siRNA sequences are as follows: rCrGrUrUrArArUrCrGrCrGrUrArUrArArUrArCrGrCrGrUA T (scramble siRNA, Cat# SR30004), rGrGrArArArUrUrArCrCrUrArUrGrUrGrCrArGrArGrGrAA T (EGFR siRNA, Cat# SR301357A), and rGrCrCrArArCrArArArGrArArArUrCrUrUrArGrArCrGrAA G (HER2 siRNA, Cat# SR301443A).

### MTT cell proliferation assay

Cells were grown in 96-well plates. Each well was seeded with 2 × 10^3^ HCT116 cells, 6 × 10^3^ HT29 cells, 5 × 10^3^ SW48 cells, or 4 × 10^3^ SW620 cells with 150 μl culture medium overnight, treated with solvent, PEPD^G278D^ (5, 25 or 250 nM), cetuximab (2.75, 27.5 or 275 nM), panitumumab (2.77, 27.7 or 277 nM), or PEPD^G278D^ plus cetuximab (250 nM each) in 200 μl medium per well for 24, 48 or 72 h, and then incubated with medium containing 9.2 mM MTT (200 μl/well) at 37 °C for 3 h. The cells were then washed with PBS and mixed with DMSO (150 μl per well), and cell density was determined by measuring the reduction of MTT to formazan spectroscopically at 570 nm using a Synergy 2 Multi-Mode Microplate Reader (BioTek). In experiments involving siRNA transfection, 2 × 10^3^ HCT116 cells or 6 × 10^3^ HT29 cells were seeded to each well of 96-well plates overnight and then transfected with 25 nM siRNA (scramble siRNA, EGFR siRNA or HER2 siRNA) as described above, followed by MTT assay.

### Immunofluorescence staining and confocal microscopy

SW48 cells were grown in chamber slides (4 × 10^4^ cells/well) overnight with or without subsequent treatment with PEPD^G278D^ (25 nM) and/or chloroquine (25 μM) for up to 6 h. The cells were then washed with ice-cold PBS, fixed with 4% paraformaldehyde for 20 min at room temperature (RT), washed again with ice-cold PBS and blocked with 1% BSA in PBS for 1 h at RT. The cells were then incubated with an EGFR antibody, a His tag antibody for detection of PEPD^G278D^, and/or a LAMP1 antibody overnight at 4 °C, washed with PBS, incubated with a secondary antibody conjugated to Alexa-Fluor 488 or Alexa-Fluor 647 for 1 h at RT and washed again with PBS. The cells were then examined with a BZ-X700 fluorescence microscope (Keyence) with a S PL FL ELWD ADM 40xC objective. Merged images from Z-stack were organized using the ImageJ software (NIH Image).

### PI3K activity assay

PI3K activity was measured using a PI3-Kinase Activity ELISA Kit from Echelon Biosciences, following manufacturer’s instruction. Briefly, PI3K was pulled down from whole cell lysates using an antibody for PI3K/p85. Each sample was prepared from approximately 1 × 10^6^ cells. The entire immunoprecipitate from each sample was mixed with 30 μl of KBZ reaction buffer, which was then mixed with 30 μl of 10 μM PI(4,5)P2 substrate and incubated for 2 h at 37 °C. The kinase reaction was terminated by adding 90 μl of kinase stop solution to each reaction solution, and 60 μl of each mixture was transferred together with 60 μl of PIP3 detector to a well in the incubation plate. After incubation at RT for 1 h, 100 μl per sample from the incubation plate was transferred to the detection plate and incubated for 1 h at RT. The detection plate was washed with TBST, incubated with the HRP-conjugated secondary detector for 30 min, washed again with TBST, and the immobilized HRP was measured by a standard colorimetric assay, using 3,3′,5,5′-tetramethylbenzedine as a substrate and a Synergy 2 Multi-Mode Microplate Reader to record absorbance.

### RAS activity assay

Ras activity in cell lysates was measured using a Ras Activation ELISA Kit from Cell Biolabs. Briefly, approximately 5 × 10^6^ cells were mixed with 0.5 ml lysis buffer. Cell lysates were cleared by centrifugation at 14,000 g for 10 min at 4 °C, and 250 μl per sample was mixed with 10 μl of 0.5 M EDTA with or without 5 μl of either 100x GTPγS or 100x GDP, which was incubated at 37 °C for 30 min with agitation and then mixed with 33 μl of 1 M MgCl_2_. The mixture after appropriate dilution was transferred at 100 μl per sample to a well in a 96-well plate immobilized with RAF-1 RAS-binding domain and incubated for 1 h at RT. After washing the wells 5 times with wash buffer, 100 μl of an anti-pan-RAS antibody was added to each well and incubated for 1 h at RT. After another round of wash, 100 μl of a secondary antibody-HRP conjugate was added to each well and incubated for 1 h at RT. After yet another round of wash, 100 μl of substrate solution was added to each well and incubated at RT for 20 min, followed by addition of 100 μl of stop solution to each well. Absorbance at 450 nm in each well was recorded by a Synergy 2 Multi-Mode Microplate Reader.

### Measurement of plasma PEPD^G278D^ concentration

Plasma PEPD^G278D^ concentration was measured by ELISA as previously reported [26]. Briefly, 96-well ELISA plates were coated with 100 μl/well of a PEPD antibody (mouse monoclonal, against amino acids 101–305, sc-390,042) overnight at 4 °C. The plates were then washed three times with PBST and incubated with 200 μl/well of a blocking buffer for at least 2 h at RT. The plates were washed again with PBST and incubated with 100 μl/well of a PEPD standard or a sample, which were appropriately diluted, for 2 h at RT. After another round of wash with PBST, each well was incubated with 100 μl of a detection antibody (an anti-PEPD rabbit polyclonal, ab-86,507) for 2 h at RT. After another round of wash with PBST, 100 μl of a secondary antibody-HRP conjugate was added to each well, followed by 1 h incubation at RT. The plates were washed again with PBST three times, and each well was then incubated with 100 μl of a HRP substrate solution. After adequate color development, 100 μl of stop solution was added to each well, and absorbance at 450 nm was recorded by a Synergy 2 Multi-Mode Microplate Reader. Purified recombinant PEPD^G278D^ was used as a standard.

### Measurement of AREG and HB-EGF

Concentrations of AREG and HB-EGF in tumor tissues were measured using the Human Amphiregulin / AREG ELISA Kit PicoKine and HB-EGF ELISA Kit, following the manufacturer’s instruction. Briefly, 100 μl of standard or tumor tissue homogenate (cleared by centrifugation) was added to a microtiter well pre-coated with anti-human AREG or anti-human HB-EGF and incubated for 2 h at RT. After washing the plate with washing buffer, 100 μl of biotinylated anti-human AREG or biotinylated anti-human HB-EGF were added to each well and incubated for 1.5 h at RT. The microtiter wells were washed 3 times with wash buffer and incubated with 100 μl/well of avidin-biotin-peroxidase complex for 40 min at RT. The microtiter wells were washed 5 times with wash buffer and incubated with 90 μl/well color developing reagent for 30 min at RT. After adding 100 μl of stop solution to each well, absorbance at 450 nm was recorded by a Synergy 2 Multi-Mode Microplate Reader.

### Preparation of cell lysates and tumor tissue homogenates

To prepare whole cell lysates, cells were washed with PBS twice, mixed with 1x cell lysis buffer from Cell Signaling Technology supplemented with 2 mM PMSF and a protease inhibitor cocktail from Roche Applied Science, placed on ice for 10 min, sonicated at 0–4 °C to enhance cell lysis using a Branson Model 450 sonifier, and finally centrifuged at 13,000 g for 10 min at 4 °C, and the supernatant fraction is collected as whole cell lysate. Tumor samples were mixed with RIPA buffer (25 mM Tris-HCl, PH7.6, 150 mM NaCl, 1% Nonidet P-40, 1% sodium deoxycholate, and 0.1% SDS), which was supplemented with 2 mM PMSF, the protease inhibitor cocktail mentioned above, phosphatase inhibitor cocktail 2 and phosphatase inhibitor cocktail 3 from Sigma-Aldrich at 14.3 μl buffer per mg tissue, and homogenized in a Dounce homogenizer. The homogenates were cleared by centrifugation at 13,000 g for 15 min at 4 °C.

### Western blotting and immunoprecipitation (IP)

Protein concentrations in all samples were measured using the BCA Assay Kit. For Western blotting, each sample was mixed with 4x loading dye, heated for 5 min at 95 °C, and resolved by SDS-PAGE (8–12.5%). Proteins were transferred to polyvinylidene fluoride membrane, probed with specific antibodies, and detected using either Luminata Classico (Millipore) or Luminata Cresendo (Millipore). For IP, cell lysates (0.5 mg protein/sample) were incubated with a desired antibody overnight at 4 °C, followed by incubation of 500 μl sample with 30 μl G-Sepharose beads (2 mg/ml) for 1 h at RT. The beads were washed three times with IP buffer, suspended in 2x SDS loading buffer, boiled for 5 min, and analyzed by Western blotting.

### Mouse study

SCID mice (C.B.17 SCID) were bred by the Laboratory Animal Shared Resource at Roswell Park Comprehensive Cancer Center. Male mice at 7–8 weeks of age were used. All mouse experiments were approved by the Institutional Animal Care and Use Committee at Roswell Park Comprehensive Cancer Center under protocol 1022 M. We established subcutaneous tumors by inoculating 1 × 10^6^ HCT116 cells, 4 × 10^6^ HT29 cells, or 2 × 10^6^ SW48 cells to the flank of each mouse in 100 μl of serum free medium. Colon patient-derived xenograft (PDX) 14650 was established from a liver metastatic lesion in a patient treated at Roswell Park Comprehensive Cancer Center. Tumor fragments (~ 20 mm^3^) from donor mice were implanted into the flank of each mouse subcutaneously using a trocar. Orthotopic colon tumors in mice were established by inoculating HCT116 cells stably transfected with firefly luciferase (2 × 10^6^ cells in 50 μl of 50% serum-free medium and 50% Matrigel) to the caecum wall of each mouse. Mice in each tumor model were randomized cage-wise into treatment groups using Research Randomizer (www.randomizer.org). Subcutaneous tumor volume was measured using length x width^2^ ÷ 2. Tumor volume was measured three times each week. Orthotopic tumor growth was monitored by bioluminescence weekly. Mice were given D-luciferin (150 mg/kg) by ip and anesthetized with isoflurane, and tumor burden was measured by bioluminescence using the IVIS Spectrum In Vivo Imaging System (PerkinElmer). Drug treatment was started when significant tumor growth was detected. EP (0.5 mg/kg) was administered to mice ip once daily. PEPD^G278D^ (4 mg/kg) was administered to mice ip thrice weekly (Monday, Wednesday, Friday). Aderbasib (60 mg/kg) was administered to mice by gavage once daily. 5-FU (35 mg/kg) was administered to mice ip twice weekly (Monday and Thursday). EP, PEPD^G278D^ and 5-FU were administered to mice in PBS. Aderbasib was first dissolved in DMSO and then diluted by PBS (final 5% DMSO by volume). Each agent was administered to mice at 0.1 ml volume per 20 g body weight. When a mouse was given multiple agents on the same day, the agents were dosed at approximately 30 min intervals. The mice were closely monitored for sign of adverse effects and weighed three times each week. Mice were sacrificed 24–48 hours after the last treatment, at which point the tumors were promptly removed, snap frozen and stored at − 80 °C for later analysis. Some tumors were fixed in 10% buffered formalin, paraffin embedded, cut at 4 μm, and stained with hematoxylin and eosin (H & E) for histological analysis.

### Statistical analysis

Data were analyzed by two-sided t-test or Mann-Whitney U test for two-group comparison, or one-way analysis of variance (ANOVA) for multi-group comparisons (followed by Tukey multiple comparisons test), using GraphPad Prism 9 software. *P* value of 0.05 or lower was considered statistically significant. Sample size, mean, SD or SEM, and P value are provided in each figure legend. Each replicate represents an independent sample, not repeated measurement of the same sample.

## Results

### PEPD^G278D^ inhibits CRC cells resistant to EGFR MABs

**Fig. 1:**
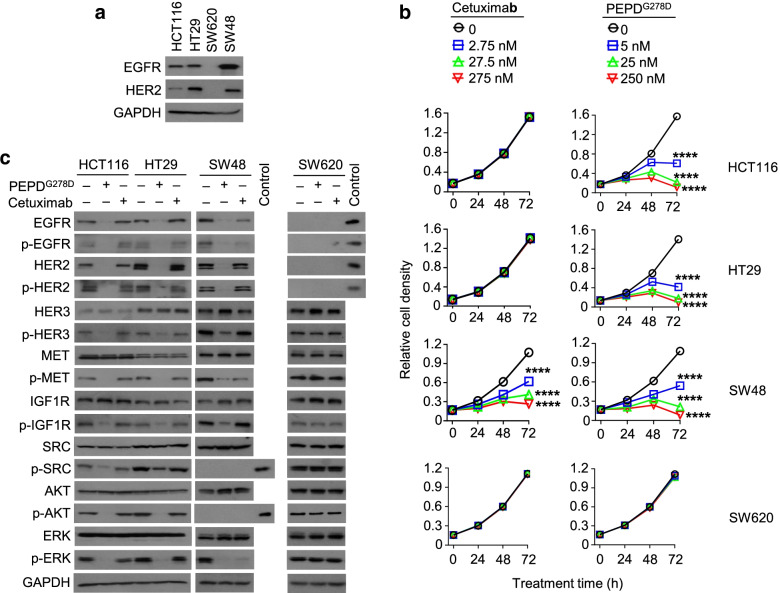
PEPD^G278D^ inhibits CRC cells resistant to cetuximab. **a** Western blotting of untreated whole cell lysates. **b** Effects of PEPD^G278D^ and cetuximab on cell growth measured by MTT assay. Each value is mean ± SD (*n* = 3). ****P < 0.0001 by one-way ANOVA, followed by Tukey test for comparison with the control. **c** Western blotting of whole cell lysates after treatment of the cells with vehicle, PEPD^G278D^ (25 nM), or cetuximab (275 nM) for 48 h. The following phosphorylation sites were measured: pY1173-EGFR, pY1221/1222-HER2, pY1328-HER3, pY1234/1235-MET, pY1131-IGF1R, pY416-SRC, pS473-AKT, and pT202/Y204-ERK. Glyceraldehyde 3-phosphate dehydrogenase (GAPDH) was measured as a loading control here and elsewhere. HCT116 cell lysates were used as a positive control for measurement of p-SRC and p-AKT in SW48 cells and for measurement of EGFR and HER2 in SW620 cells

We compared the response of four human CRC cell lines to cetuximab, panitumumab, and PEPD^G278D^, including HCT116, HT29, SW48, and SW620 cells. Both EGFR and HER2 were expressed in HCT116, HT29, and SW48 cells, but their expression levels varied greatly among the cell lines, whereas neither EGFR nor HER2 could be detected in SW620 cells (Fig. 1a). HCT116 cells carry activating mutations of KRAS (G13D) and PIK3CA (H1047R), and HT29 cells carry activating mutations of BRAF (V600E) and PIK3CA (P449T) (Suppl. Fig. 1). Mutated KRAS, BRAF and PIK3CA are widely believed to drive resistance of CRC cells to EGFR MABs by rendering the cells independent of EGFR.

Both cetuximab and panitumumab inhibited the growth of SW48 cells in a time- and concentration-dependent manner, but neither agent is active in HCT116 cells and HT29 cells (Fig. 1b and Suppl. Fig. 2a). Cetuximab was also evaluated in SW620 cells and was inactive (Fig. 1b), which is expected, since these cells do not express EGFR. PEPD^G278D^ strongly inhibited the growth of SW48, HCT116, and HT29 cells in a time- and concentration-dependent manner but was ineffective in SW620 cells (Fig. 1b). We previously showed that PEPD^G278D^ specifically targets EGFR and HER2 [19, 20, 22]. Although both PEPD^G278D^ and the EGFR MBAs inhibit SW48 cells, the former is more potent than the latter. We next analyzed EGFR, several other RTKs, including HER2, HER3, MET, IGF1R, and several key downstream signaling proteins, including AKT, ERK, and SRC. EGFR and HER2 form heterodimeric signaling units with various RTKs to diversify their oncogenic signaling [28, 29]. The growth-inhibitory activities of the EGFR MABs in SW48 cells were accompanied by decrease in expression and phosphorylation of EGFR as well as decrease in phosphorylation but not expression of MET and ERK (Fig. 1c and Suppl. Fig. 2b). p-AKT and p-SRC were undetectable in SW48 cells. Decreased phosphorylation of MET and ERK induced by the EGFR MABs in SW48 cells apparently resulted from EGFR inhibition, as neither cetuximab nor panitumumab had any effect on MET and ERK in HCT116 cells and HT29 cells in which EGFR was not inhibited as well as in SW620 cells which do not express EGFR. Cetuximab was also evaluated against HER3 and IGF1R in SW48 cells and showed no effect on the RTKs. Cetuximab showed no effect on any of the signaling proteins in HCT116 and HT29 cells (Fig. 1c). PEPD^G278D^ obliterated both expression and tyrosine phosphorylation of EGFR and HER2 in SW48, HCT116 and HT29 cells (Fig. 1c). PEPD^G278D^ is more effective than the EGFR MABs in obliterating EGFR in SW48 cells, even though cells were treated by PEPD^G278D^ at 25 nM but by the MABs at 275–277 nM. In HCT116, HT29 and SW48 cells, PEPD^G278D^ had no effect on the expression of other signaling proteins but markedly decreased their phosphorylation (Fig. 1c). PEPD^G278D^ inhibition of phosphorylation of HER3, IGF1R and MET but not their expression is consistent with its disruption of EGFR association with HER3, MET or IGF1R (Suppl. Fig. 3). We previously showed that PEPD^G278D^ also disrupts the association of HER2 with each of these RTKs in HER2-positive breast cancer cells [21]. In SW620 cells that lack EGFR and HER2, PEPD^G278D^ had no effect on the phosphorylation or expression of HER3, MET, IGF1R, SRC, AKT, and ERK (Fig. 1c). These results show that by depleting EGFR and HER2 in CRC cells, PEPD^G278D^ not only directly suppresses both RTKs but also indirectly suppresses other RTKs by disrupting their association with EGFR or HER2, thereby causing extensive inhibition of oncogenic signaling. Indeed, siRNA knockdown of EGFR or HER2 also significantly inhibited the growth of HCT116 cells and HT29 cells (Suppl. Fig. 4a-b). Our results indicate that the inability of EGFR MABs to downregulate EGFR in HCT116 cells and HT29 cells is primarily responsible for their failure to inhibit the growth of these cells, rather than compensatory signaling driven by mutated KRAS, BRAF and PIK3CA. Our results also show that PEPD^G278D^ is active in CRC cells overexpressing different levels of EGFR and HER2.

### PEPD^G278D^ abolishes RAS-ERK and PI3K-AKT signaling despite activating mutations in KRAS, BRAF, and PIK3CA

**Fig. 2:**
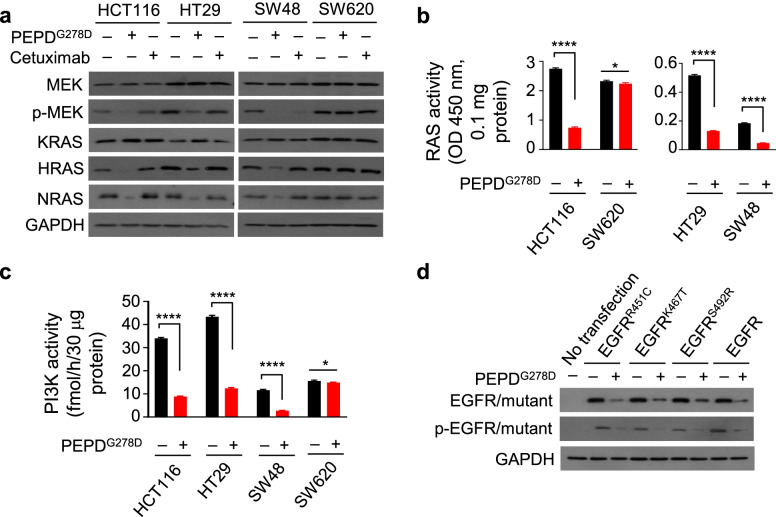
PEPD^G278D^ inhibits RAS and PI3K and targets EGFR mutants in CRC cells. **a** Western blotting of whole cell lysates from cells treated with solvent, PEPD^G278D^ (25 nM), and cetuximab (275 nM) for 48 h. **b**, **c** Ras and PI3K activities in whole cell lysates from cells treated with solvent or PEPD^G278D^ (25 nM) for 48 h. Each value is mean ± SD (*n* = 3). *P < 0.05, ****P < 0.0001 by two-tailed unpaired t test. **d** Western blotting of whole cell lysates from untreated cells or cells which were transfected with EGFR or its mutant and 24 h later treated with solvent or PEPD^G278D^ (25 nM) for 48 h. p-MEK and p-EGFR/mutant are pS217/221-MEK and pY1173-EGFR, respectively

Although HCT116 cells carry activating mutations of KRAS and PIK3CA, and HT29 cells carry activating mutations of BRAF and PIK3CA, and both cell lines are resistant to cetuximab and panitumumab, as described above, the inhibitory activities of PEPD^G278D^ in HCT116 cells and HT29 cells were similar to that in SW48 cells whose KRAS, BRAF and PIK3CA are not mutated. ERK and AKT are downstream of KRAS and PIK3CA, respectively. PEPD^G278D^ caused marked loss of phosphorylation of ERK and AKT in both HCT116 and HT29 cells (Fig. 1c). MEK is upstream of ERK, and PEPD^G278D^ also markedly decreased MEK phosphorylation in both HCT116 and HT29 cells (Fig. 2a). The loss of phosphorylation of MEK, ERK and AKT apparently resulted from PEPD^G278D^ targeting EGFR and HER2, as PEPD^G278D^ was inactive in SW620 cells and cetuximab was active only in SW48 cells (Figs. 1c and 2a).

It was previously shown that oncogenic RAS mutants regulate basal effector pathway signaling, while WT RAS in the same cells mediates signaling downstream of activated RTKs [30]. It was also shown that the gain of function of PIK3CA mutants is enabled by activated RAS or PI3K/p85-mediated binding to activated RTKs [31, 32]. Notably, only one allele of each of the KRAS and PIK3CA genes in HCT116 cells is mutated, and only one allele of each of the BRAF and PIK3CA genes in HT29 cells is mutated (Suppl. Fig. 1). Moreover, HRAS and NRAS are also expressed in both HCT116 and HT29 cells as well as in SW48 and SW620 cells (Fig. 2a). These results provide an explanation for why PEPD^G278D^ is able to strongly inactivate MEK, ERK and AKT in HCT116 cells and HT29 cells. We also found that PEPD^G278D^ strongly downregulates the expression of both HRAS and NRAS in HCT116, HT29 and SW48 cells but not in SW620 cells, whereas cetuximab had no effect on the expression of HRAS and NRAS in any of the cell lines (Fig. 2a). Since cetuximab only targets EGFR, the above results suggested that downregulation of HRAS and NRAS by PEPD^G278D^ might result from HER2 depletion. Indeed, siRNA silence of HER2 but not EGFR resulted in loss of HRAS and NRAS (Suppl. Fig. 4a). However, neither PEPD^G278D^ nor cetuximab regulated the expression of WT or mutated KRAS (Fig. 2a).

Consistent with the changes in the signaling molecules described above, total RAS and PI3K activities were strongly inhibited by PEPD^G278D^ in HCT116 and HT29 cells as well as in SW48 cells but not in SW620 cells (Fig. 2b-c). There was little difference among HCT116, HT29 and SW48 cells with regard to percentage of inhibition of RAS and PI3K by PEPD^G278D^, as RAS activity was inhibited by 73% in HCT116 cells and 75% in both HT29 and SW48 cells, and PI3K activity was inhibited by 74% in HCT116 cells, 71% in HT29 cells, and 76% in SW48 cells. Notably, basal RAS activity is much higher in HCT116 and SW620 cells than in HT29 and SW48 cells, consistent with HCT116 cells carrying KRAS^G12D^ (Suppl. Fig. 1) and SW620 cells carrying KRAS^G12V^ [27], and basal PI3K activity is much higher in HCT116 and HT29 cells than in SW48 and SW620, consistent with HCT116 cells carrying PIK3CA^H1047R^ and HT29 cells carrying PIK3CA^P449T^ (Suppl. Fig. 1). Also, the remaining RAS and PI3K activities after PEPD^G278D^ treatment were still higher in HCT116 and HT29 cells than in SW48 cells. Collectively, our results show that PEPD^G278D^ strongly inhibits the RAS-MEK-ERK and PI3K-AKT signaling pathways by depleting EGFR and HER2, even if CRC cells harbor activating mutations of KRAS, BRAF and/or PIK3CA. Our results also indicate that PEPD^G278D^ accomplishes this feat by abolishing both canonical function (tyrosine kinase) and non-canonical function (scaffolding – heterodimerization with other RTKs) of EGFR and HER2 as well as abolishing HER2 regulation of HRAS and NRAS.

### PEPD^G278D^ also targets EGFR mutants that occur in CRC patients

While EGFR is not mutated in SW48, HCT116 and HT29 cells [27], several acquired mutations in the extracellular domain of EGFR have been reported in CRC patients following cetuximab treatment, including R451C, K467T, and S492R, each of which prevents cetuximab binding and confers resistance to cetuximab [33, 34]. EGFR^R451C^ and EGFR^K467T^ also bind poorly to panitumumab [33]. However, these mutations locate far from the site (amino acids #166–310) to which PEPD^G278D^ binds [20]. Because SW620 cells do not express EGFR, we transfected each EGFR mutant as well as WT EGFR into these cells and then treated the cells with solvent or PEPD^G278D^ (25 nM for 48 h). Each EGFR mutant was strongly downregulated by PEPD^G278D^, showing loss of both expression and phosphorylation, and the extent of downregulation of each mutant by PEPD^G278D^ is very similar to that of WT EGFR (Fig. 2d). Thus, mutations in EGFR which occur in CRC patients do not interfere with PEPD^G278D^ targeting of EGFR.

### EGFR ligands slow PEPD^G278D^ induction of EGFR internalization and lysosomal degradation

**Fig. 3:**
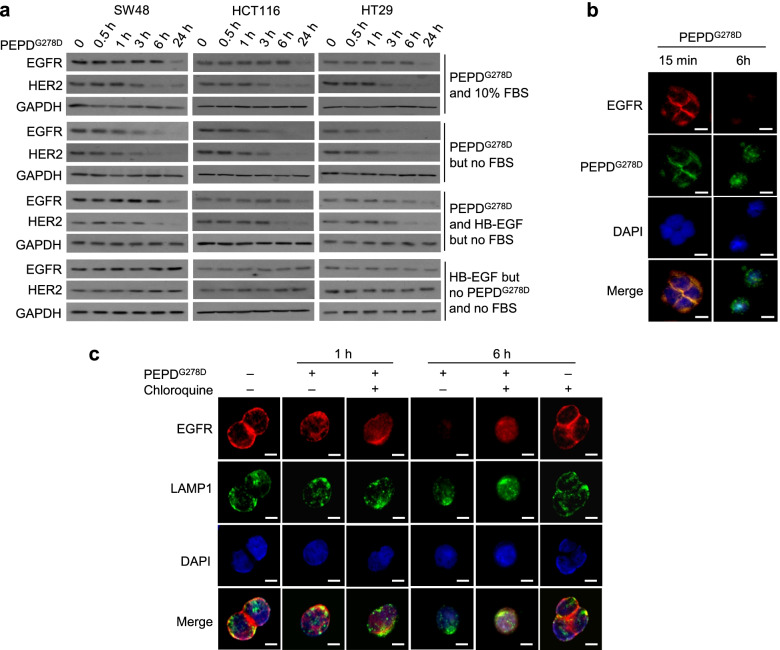
PEPD^G278D^ induction of EGFR degradation is slowed by EGFR ligands. **a** Western blotting of whole cell lysates from untreated cells, cells treated with PEPD^G278D^ (25 nM) for different times in medium containing 10% FBS, no FBS, or HB-EGF (20 ng/ml) without FBS, or from cells treated with HB-EGF (20 ng/ml) without PEPD^G278D^ and FBS. **b** Confocal fluorescence staining of EGFR, PEPD^G278D^ and nuclei (DAPI) in SW48 cells treated with PEPD^G278D^ (25 nM) for 15 min or 6 h. **c** Confocal fluorescence staining of EGFR, LAMP1 and nuclei (DAPI) in SW48 cells with no treatment, or treated with PEPD^G278D^ (25 nM) and/or chloroquine (25 μM) for 1 or 6 h. Scale bars in **b**, **c**: 10 μm

PEPD^G278D^ causes depletion of both EGFR and HER2, but HER2 depletion was much faster than that of EGFR in cells cultured in medium with 10% serum. In HCT116, HT29, and SW48 cells, HER2 level decreased markedly after 3 h of PEPD^G278D^ treatment, whereas EGFR level showed no decrease even after 6 h of PEPD^G278D^ treatment, although it showed profound decrease at 24 h (Fig. 3a). However, if the cells were cultured in serum-free medium, both EGFR and HER2 showed marked decrease after 3 h treatment with PEPD^G278D^ (Fig. 3a). We previously showed that epidermal growth factor (EGF), a high affinity EGFR ligand, competes with PEPD for binding to EGFR [26]. Notably, EGFR ligands bind to subdomains 1 and 3 in EGFR extracellular domain [35, 36], whereas PEPD^G278D^ binds to subdomain 2 in EGFR [20]. Adding HB-EGF, another high affinity EGFR ligand, to culture medium without serum mimicked the effect of serum on EGFR depletion induced by PEPD^G278D^, while HB-EGF itself did not modulate the expression of EGFR or HER2 (Fig. 3a). No HER2 ligand, other than PEPD or PEPD^G278D^, is known. These results suggest that EGFR ligands from serum interfere with PEPD^G278D^ targeting of EGFR. We also found that cetuximab attenuates the growth-inhibitory activity of PEPD^G278D^ in both HCT116 cells and HT29 cells (Suppl. Fig. 5a), which likely resulted from cetuximab competing with PEPD^G278D^ for EGFR binding. Notably, cetuximab binds to extracellular subdomain 3 of EGFR [37].

We previously showed that PEPD^G278D^ induces HER2 internalization and lysosomal degradation [21]. Here, we show that PEPD^G278D^ also induces EGFR internalization and lysosomal degradation. We focused on SW48 cells, taking advantage of their high EGFR level. SW48 cells were cultured in serum-free medium. PEPD^G278D^ binding to EGFR and subsequent EGFR trafficking were analyzed by immunofluorescence staining and confocal microscopy. PEPD^G278D^ bound abundantly to cell membrane and colocalized with EGFR after 15 min of treatment, but at 6 h, neither PEPD^G278D^ nor EGFR remained on cell membrane, with residual amount of PEPD^G278D^ but no EGFR detected intracellularly (Fig. 3b). Next, cells were treated with PEPD^G278D^ and/or chloroquine, the latter of which is a lysosome inhibitor. In the absence of chloroquine, PEPD^G278D^ induced EGFR internalization, and the internalized EGFR colocalized with LAMP1, a lysosome marker, but at 6 h of treatment, almost no EGFR could be detected (Fig. 3c). However, chloroquine blocked EGFR degradation induced by PEPD^G278D^ (Fig. 3c). Collectively, our results show that PEPD^G278D^ induces EGFR internalization and degradation in the lysosome but EGFR ligands slow this process by interfering with PEPD^G278D^ binding to EGFR.

### PEPD^G278D^ fails to inhibit tumors that overexpress a high-affinity EGFR ligand

**Fig. 4:**
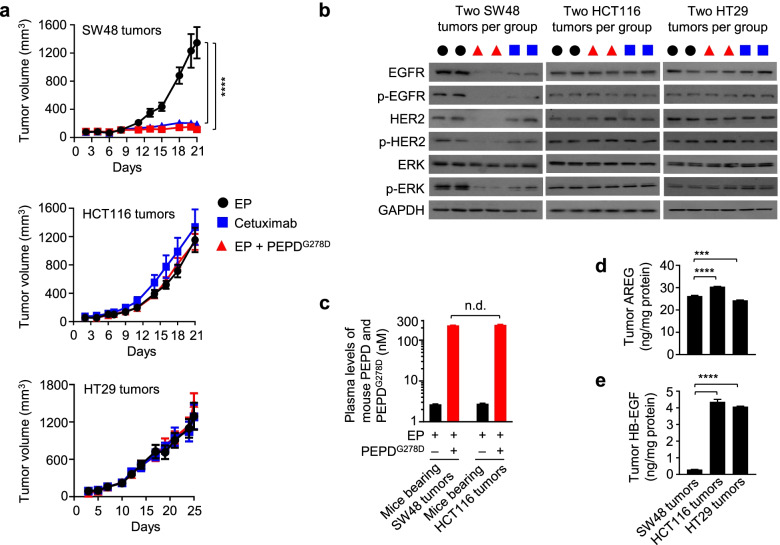
The antitumor activities of cetuximab and PEPD^G278D^ in vivo correlate with EGFR downregulation. **a** Mice bearing subcutaneous tumors were treated with EP daily (SW48 tumors: days 2–20; HCT116 tumors: days 3–20; HT29 tumors: days 8–24; *n* = 12), cetuximab twice weekly (SW48 tumors: days 8–20; HT116 tumors: days 7–20; HT29 tumors: 10–24; *n* = 12–14), or EP daily plus PEPD^G278D^ thrice weekly (SW48 tumors: days 8–20; HCT116 tumors: days 7–20; HT29 tumors: days 10–24; *n* = 12–14). EP, cetuximab, and PEPD^G278D^ were administered ip at 0.5, 15, and 4 mg/kg per dose, respectively. Group average tumor sizes were 107–111 mm^3^ (SW48 tumors), 100–129 mm^3^ (HCT116 tumors), and 219–229 mm^3^ (HT29 tumors) at the beginning of treatment. Each value is mean ± SEM. ****P < 0.0001 by one-way ANOVA, followed by Tukey test for comparison with the control. **b** Western blotting of tumor homogenates (2 tumors per group). Tumors were harvested 24 h after the final treatment. See Fig. 1 legend for protein phosphorylation sites. **c** Plasma levels of mouse PEPD and PEPD^G278D^ at 24 h after final PEPD^G278D^ treatment. Each value is mean ± SD (*n* = 3). n.d., not significant by two-tailed unpaired t test. **d, e** Levels of soluble AREG and HB-EGF in control tumors. Each value is mean ± SD (*n* = 3). ***P < 0.001, ****P < 0.0001, by two-tailed unpaired t test

We next compared the antitumor activities of PEPD^G278D^ and cetuximab in vivo. PEPD^G278D^ is degraded in vivo by coagulation proteases, but EP, a clinically used anticoagulant, inhibits PEPD^G278D^ degradation [38]. EP itself has no antitumor activity but combining EP with PEPD^G278D^ allows therapeutically relevant plasma concentrations of PEPD^G278D^ to be achieved for inhibition of tumors overexpressing EGFR and/or HER2 [20, 22]. We inoculated human CRC cells to immunocompromised mice subcutaneously, and the tumor-bearing mice were randomized for treatment with EP, EP plus PEPD^G278D^, or cetuximab. Based on previous studies, EP was administered to the mice at 0.5 mg/kg daily by intraperitoneal injection (ip); PEPD^G278D^ was administered at 4 mg/kg ip three times weekly; and cetuximab was administered at 15 mg/kg ip twice weekly. Both PEPD^G278D^ and cetuximab strongly inhibited the growth of SW48 tumors and at the end of treatment inhibiting tumor growth by 91.5 and 85.8%, reactively, but neither agent inhibited the growth of HCT116 tumors and HT29 tumors (Fig. 4a). Escalating PEPD^G278D^ to 8 mg/kg did not inhibit tumor growth either (Suppl. Fig. 5b). Tumors were collected 24 h after the final dose of each agent and select signaling proteins were analyzed. In SW48 tumors, PEPD^G278D^ decreased the expression and phosphorylation of both EGFR and HER2, while cetuximab only decreased the expression and phosphorylation of EGFR, and both agents also decreased ERK phosphorylation (Fig. 4b). Similar results were shown in cultured SW48 cells as described before. However, neither PEPD^G278D^ nor cetuximab had any effect on EGFR, HER2 and ERK in HCT116 and HT29 tumors (Fig. 4b). Yet, in cultured HCT116 and HT29 cells as described before, while cetuximab was inactive, PEPD^G278D^ strongly reduced the expression and phosphorylation of both EGFR and HER2 and decreased ERK phosphorylation.

Lack of inhibitory activity of PEPD^G278D^ in HCT116 tumors and HT29 tumors was not due to lack of PEPD^G278D^ delivery, as plasma concentrations of PEPD^G278D^ were high and similar in mice bearing SW48 tumors and HCT116 tumors (Fig. 4c). We analyzed all seven known EGFR ligands (soluble form) in the tumor tissues but detected only AREG and HB-EGF. AREG is a low affinity EGFR ligand, and its affinity for EGFR is approximately 50 fold lower than that of HB-EGF [39]. AREG level was high in all three types of tumors (Fig. 4d). HB-EGF level was 13.6–14.6 fold higher in HT29 and HCT116 tumors than in SW48 tumors (Fig. 4e). This suggests that excessive tumor-generated HB-EGF might prevent PEPD^G278D^ from binding to EGFR, and also suggests that EGFR signaling remains important to the tumors carrying activating mutations of KRAS, BRAF and/or PIK3CA. PEPD^G278D^ also failed to downregulate HER2 in tumors expressing high level of HB-EGF. HB-EGF does not bind to HER2, and we showed previously that PEPD^G278D^ disrupts the HER2-EGFR heterodimer even when EGF is bound to EGFR [22]. However, it is possible that without inhibiting EGFR, the impact of PEPD^G278D^ on HER2 may be negated by rapid tumor growth.

### Aderbasib restores the antitumor activity of PEPD^G278D^ in tumors overexpressing HB-EGF

**Fig. 5:**
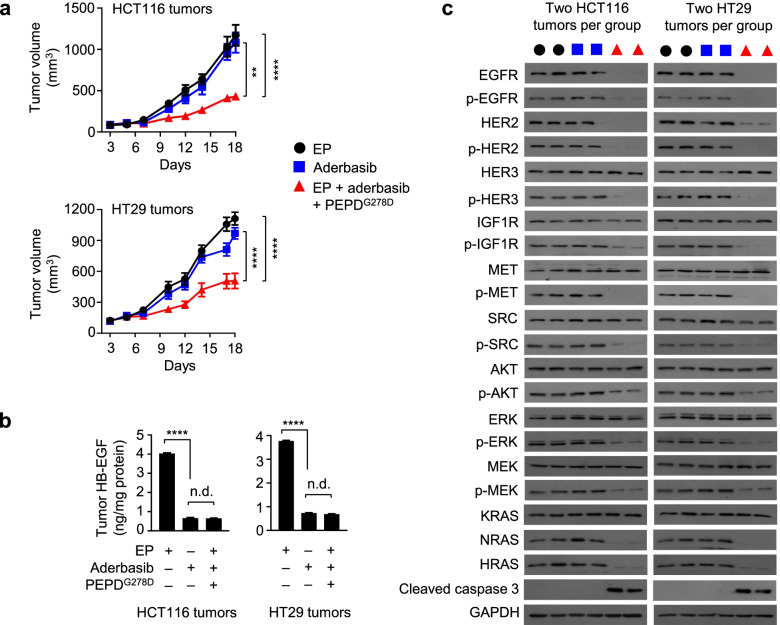
Aderbasib restores the antitumor activity of PEPD^G278D^ by inhibiting HB-EGF shedding. **a** Mice bearing subcutaneous tumors were randomized to EP treatment (*n* = 12–16), aderbasib treatment (*n* = 12), or treatment with EP plus aderbasib and PEPD^G278D^ (*n* = 12). Mice were treated with EP daily (HCT116 tumors: days 2–17; HT29 tumors: days 3–17), aderbasib daily (HCT116 tumors: days 4–17; HT29 tumors: days 4–17), and PEPD^G278D^ thrice weekly (HCT116 tumors: days 5–17; HT29 tumors: days 5–17). EP and PEPD^G278D^ were administered to mice by ip at 0.5, and 4 mg/kg per dose, respectively. Aderbasib was administered to mice by gavage at 60 mg/kg per dose. Group average tumor sizes were 92–110 mm^3^ (HCT116 tumors) and 153–172 mm^3^ (HT29 tumors) at the beginning of treatment. Each value is mean ± SEM. **P < 0.01, ****P < 0.001, by one-way ANOVA, followed by Tukey test. **b** Tumor levels of soluble HB-EGF. Each value is mean ± SD (*n* = 3). ****P < 0.0001 by one-way ANOVA, followed by Tukey test. **c** Western blotting of tumor homogenates (2 tumors per group). Tumors were harvested 24 h after final treatment. See Fig. 1 and Fig. 2 legends for protein phosphorylation sites

Aderbasib inhibits the shedding of all EGFR ligands by inhibiting ADAM10 and ADAM17 as mentioned before. We evaluated the antitumor activity of aderbasib as a single agent or in combination with EP plus PEPD^G278D^ in HCT116 and HT29 tumors. Tumor-bearing mice were treated with EP, aderbasib, or the combination of aderbasib with EP and PEPD^G278D^. As in previous experiments, EP was administered at 0.5 mg/kg ip daily, and PEPD^G278D^ was administered at 4 mg/kg ip three times weekly. Aderbasib was administered at 60 mg/kg by gavage daily. In HCT116 and HT29 tumors, aderbasib alone was ineffective, but combining aderbasib with EP plus PEPD^G278D^ inhibited tumor growth by 63.3 and 54.4% respectively at the end of treatment (Fig. 5a). The combination treatment was less effective when aderbasib was reduced to 30 mg/kg and became ineffective when it was reduced to 15 mg/kg (Suppl. Fig. 6a-c). Mice treated with aderbasib alone or the combination regimen did not show signs of toxicity.

Aderbasib caused marked decrease in soluble HB-EGF level in the tumor tissues (Fig. 5b). In both tumor models, neither EP nor aderbasib had any effect on the expression or phosphorylation of the proteins analyzed, but combining aderbasib with EP and PEPD^G278D^ caused profound loss of both expression and phosphorylation of EGFR and HER2, loss of phosphorylation but not expression of all other RTKs and downstream signaling proteins analyzed, including HER3, IGF1R, MET, SRC, AKT, ERK, and MEK, loss of expression of NRAS and HRAS, and activation of caspase 3 (Fig. 5c). These results show that by blocking shedding of HB-EGF from tumor cells, aderbasib enables PEPD^G278D^ to engage its targets and to exert its antitumor activity. Although not measured, aderbasib probably also blocked the shedding of AREG from the tumor cells.

Notably, in tumors treated by the triple combination (EP, aderbasib and PEPD^G278D^), low levels of p-AKT, p-ERK and p-MEK remained despite profound loss of both expression and phosphorylation of EGFR and HER2 induced by PEPD^G278D^, suggesting that mutated PIK3CA, KRAS and BRAF may sustain a low level of signaling despite depletion of EGFR and HER2. Likewise, in cultured HCT116 and HT29 cells, despite profound loss of both EGFR and HER2 and marked inhibition of both RAS and PI3K activities by PEPD^G278D^, residual RAS and PI3K activities remain as mentioned before.

### Adding 5-FU to the PEPD^G278D^-based combination treatment enhances therapeutic outcome

**Fig. 6:**
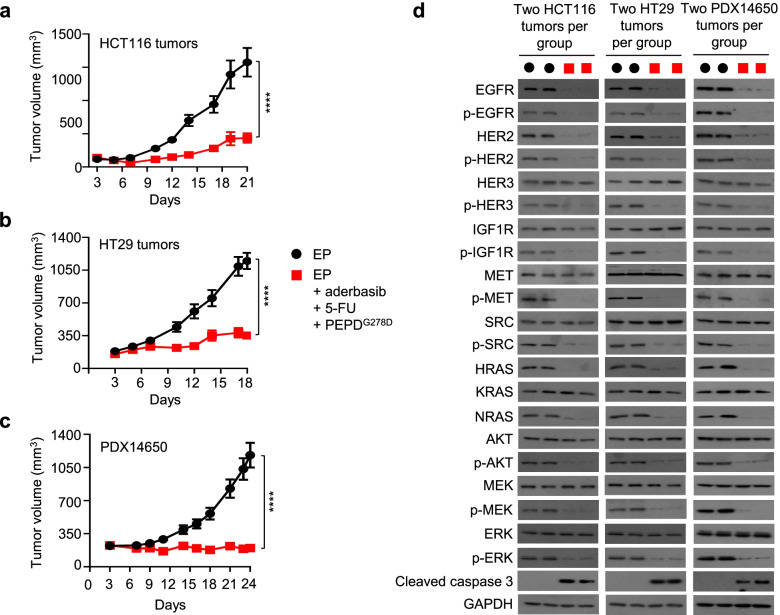
5-FU enhances the therapeutic outcome of the PEPD^G278D^-centered combination treatment. **a-c** Mice bearing subcutaneous tumors were treated with EP daily (HCT116 tumors: days 3–20; HT29 tumors: days 3–17; PDX14650: days 4–23), or EP daily plus aderbasib daily (HCT116 tumors: days 4–20; HT29 tumors: days 4–17; PDX14650: days 6–23) plus PEPD^G278D^ thrice weekly (HCT116 tumors: days 5–19; HT29 tumors: days 5–17; PDX14650: days 7–23) plus 5-FU every 3–4 days (HCT116 tumors: days 6–20; HT29 tumors: days 6–17; PDX14650: days 8–23). EP, PEPD^G278D^, and 5-FU were administered to mice by ip at 0.5, 4 and 35 mg/kg per dose, respectively. Aderbasib was administered to mice by gavage at 60 mg/kg per dose. Group average tumor volumes were 59–64 mm^3^ (HCT116 tumors), 153–185 mm^3^ (HT29 tumors), and 193–226 mm^3^ (PDX14650) at the beginning of treatment. Each value is mean ± SEM (*n* = 13–16). ****P < 0.0001 by two-tailed unpaired t test. **d** Western blotting of tumor homogenates (2 tumors per group). Tumors were harvested 24 or 48 h after final PEPD^G278D^ treatment. See Fig. 1 and Fig. 2 legends for protein phosphorylation sites

Because 5-FU, an antimetabolite, is commonly used in CRC treatment, we asked whether combining 5-FU with the PEPD^G278D^-based combination regime described above enhances treatment outcome. Thus, tumor-bearing mice were treated with EP, or the combination of EP, 5-FU, aderbasib and PEPD^G278D^. As in other experiments, EP was administered at 0.5 mg/kg ip daily, PEPD^G278D^ was administered at 4 mg/kg ip three times weekly, and aderbasib was administered by gavage at 60 mg/kg daily. 5-FU was administered to mice at 35 mg/kg ip once every 3–4 days, which was not toxic in a dose-finding experiment. We first evaluated the combination regimen in mice bearing subcutaneous HCT116 and HT29 tumors. The combination treatment was highly effective against both types of tumors, inhibiting tumor growth by 72.4% (HCT116 tumors) and 69.4% (HT29 tumors) at the end of treatment (Fig. 6a-b), which is more efficacious than the combination minus 5-FU as described before. 5-FU as a single agent or 5-FU in combination with EP and PEPD^G278D^ without aderbasib was ineffective (Suppl. Fig. 7). The combination regimen was also evaluated in a CRC PDX (PDX14650) which harbors KRAS^G12D^ (homozygous) and generates high level of HB-EGF (Suppl. Fig. 8). The combination regime markedly decreased tumor soluble HB-EGF level (Suppl. Fig. 8) and inhibited tumor growth by 83.2% at the end of treatment (Fig. 6c). Adverse effects were not detected in the mice in any of the tumor models. Tumors in the different models were collected 24 or 48 h after final treatment. In all the tumor models, the combination treatment caused profound loss in both expression and phosphorylation of EGFR and HER2, loss of phosphorylation but not expression of all other RTKs and downstream signaling proteins measured, including HER3, IGF1R, MET, SRC, AKT, MEK, ERK, loss of expression of HRAS and NRAS, and activation of caspase 3 (Fig. 6d). These results are similar to that obtained from tumors treated by aderbasib plus EP and PEPD^G278D^ and from cultured cells treated by PEPD^G278D^ as a single agent, as described before. Thus, 5-FU enhances tumor inhibition when combined with the other agents but does not interfere with the inhibition of oncogenic signaling by PEPD^G278D^ and does not interfere with aderbasib inhibition of EGFR ligand shedding by tumor cells. 5-FU is known to exert its antitumor activity by causing misincorporation of fluoronucleotides into RNA and DNA and inhibiting the nucleotide synthetic enzyme thymidylate synthase.

**Fig. 7:**
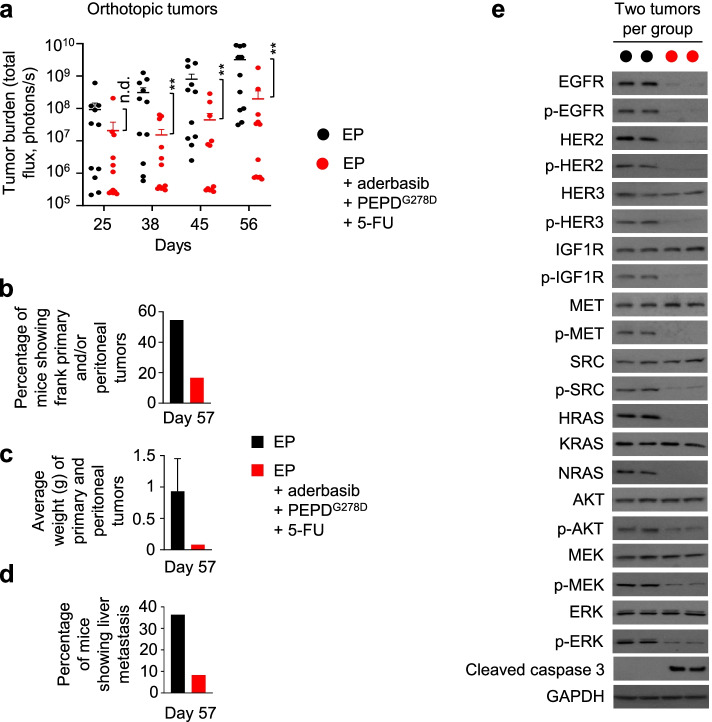
5-FU plus PEPD^G278D^-based combination treatment inhibits orthotopic CRC. **a** Tumor burden measured by bioluminescence imaging. Tumor-bearing mice were treated by EP daily (days 22–56), or EP daily plus aderbasib daily (days 26–56) plus PEPD^G278D^ thrice weekly (days 27–55) plus 5-FU every 3–4 days (days 28–56). EP, PEPD^G278D^ and 5-FU were administered to mice by ip at 0.5, 4 and 35 mg/kg per dose, respectively. Aderbasib was administered to mice by gavage at 60 mg/kg per dose. Each value is mean ± SEM (*n* = 11–12). **P < 0.01 by Mann-Whitney U test. **b-d** Percentage of mice showing frank primary tumors, average tumor weight, and percentage of mice showing liver metastasis at the end of experiment. **e** Western blotting of tumor homogenates (2 tumors per group). Tumors were harvested on day 57. See Fig. 1 and Fig. 2 legends for protein phosphorylation sites

We also evaluated the combination regimen in mice bearing orthotopic HCT116 tumors. HCT116 cells stably expressing firefly luciferase were inoculated to the cecum of mice. Tumor growth was monitored by bioluminescence imaging (Suppl. Fig. 9a). Mice were randomized to EP or the combination regimen. Treatments with EP, aderbasib, PEPD^G278D^ and 5-FU were the same as described before and were started on 22, 26, 27 and 28 days after cell inoculation, respectively. Tumor burden was not significantly different between the control and combination treatment group at the beginning of treatment (day 25), but tumor burden became significantly and consistently lower in the combination treatment group (Fig. 7a). The mean tumor bioluminescence intensity in the combination treatment group was consistently nearly 2 orders of magnitude lower than that in the control. The experiment was terminated on day 57, when several mice in the control group became moribund. Necropsy showed primary tumors in the cecum, local metastasis (peritoneal tumors), and liver metastasis. However, macroscopic tumors were found only in mice showing bioluminescence signals of > 3.2 × 10^8^ photons per second. Six of the 11 mice in the control (54.6%) and 2 of the 12 mice in the combination treatment (16.7%) showed cecum and/or peritoneal tumors (Fig. 7b). Average tumor weight (cecum and peritoneal tumors) in the combination treatment was only 8.8% of that in the control (Fig. 7c). Four of the 11 mice (36.4%) in the control showed liver metastasis, but only 1 of the 12 mice (8.3%) in the combination treatment showed liver metastasis (Fig. 7d). Representative primary and metastatic tumors are shown in Suppl. Fig. 9b. All tumors were verified by histological analysis, and representative images are shown in Suppl. Fig. 9c. Adverse effects of the treatments were not detected. As in other experimental models described before, the combination treatment caused profound loss in both expression and phosphorylation of EGFR and HER2, loss of phosphorylation but not expression of HER3, IGF1R, MET, SRC, AKT, MEK and ERK, loss of expression of HRAS and NRAS, and activation of caspase 3 (Fig. 7e).

## Discussion

It is widely believed that activating mutations of KRAS, BRAF and PIK3CA confer CRC resistance to cetuximab and panitumumab by driving EGFR-independent oncogenic signaling [10, 40, 41]. However, our present study shows that activating mutations of these genes do not render EGFR irrelevant in CRC cells and that resistance to the MABs stems primarily from their inability to downregulate EGFR. We show that 1) the growth-inhibitory activities of both cetuximab and panitumumab in CRC cells correlate with EGFR downregulation, 2) PEPD^G278D^, which induces the degradation of both EGFR and HER2, strongly inhibits the growth of CRC cells that are resistant to the EGFR MABs and carry activating mutations in KRAS, BRAF and/or PIK3CA, 3) PEPD^G278D^ strongly inhibits RAS-ERK signaling and PI3K-AKT signaling in CRC cells carrying mutated KRAS, BRAF and/or PIK3CA, 4) knockdown of EGFR or HER2 by siRNA also inhibits CRC cells resistant to the MABs, and 5) the CRC tumor models in the present study overexpress HB-EGF, despite harboring activating mutations of KRAS, BRAF and/or PIK3CA, suggesting that EGFR signaling remains important in these cells. These results are consistent with previous findings that EGFR downregulation after treatment with cetuximab or panitumumab predicts the antitumor effect in CRC [42] and that HER2 amplification confers resistance to anti-EGFR therapy in CRC [8]. Failure to downregulate EGFR by cetuximab and panitumumab in CRC may be common, as pre-treatment tumor EGFR expression level does not correlate with clinical response to the MABs [4, 43]. It is poorly understood as to why the EGFR MAbs downregulate EGFR in some CRC cells but fail to do so in other CRC cells. One study shows that EGFR insensitivity to cetuximab results from dysregulation of EGFR internalization and degradation involving CBL, an E3 ligase [44]. Another study shows that EGFR methylation in its extracellular domain renders EGFR insensitive to cetuximab [45].

PEPD^G278D^ is a promising agent for combating drug resistance in CRC. PEPD^G278D^ depletes both EGFR and HER2 by binding to their extracellular domain and inducing their internalization and degradation in lysosomes. PEPD^G278D^ is also effective against EGFR mutants that occur in CRC patients (R451C, K467T, and S492R). By depleting EGFR and HER2, PEPD^G278D^ also inactivates other RTKs that heterodimerize with EGFR or HER2. We examined three RTKs, including HER3, IGF1R, and MET, and all of them were inactivated by PEPD^G278D^ in CRC cells and tumors due apparently to disruption of their association with EGFR or HER2. Other RTKs that form heterodimeric signaling units with EGFR or HER2 may also be inactivated by PEPD^G278D^ in a similar manner. EGFR family members are known to heterodimerize promiscuously with other RTKs [28, 29]. Moreover, PEPD^G278D^ also downregulates HRAS and NRAS in CRC cells, resulting from HER2 depletion. It is also important to note that PEPD^G278D^ induces the degradation of EGFR and HER2 when they are overexpressed as in cancer cells but not when they are expressed low as in normal cells [21]. This selectivity apparently is related to its receptor-binding mode, as PEPD^G278D^ cross-links two EGFR monomers or two HER2 monomers [19, 21, 22], which requires the presence of high levels of the RTKs on cell membrane. Adverse effects of PEPD^G278D^ have not been detected in previous [20–22] and present animal studies.

PEPD^G278D^ is highly active in tumors overexpressing low-affinity EGFR ligand AREG. However, high-affinity EGFR ligands compete with PEPD^G278D^ for EGFR binding, and high level of HB-EGF in tumors blocks target engagement by PEPD^G278D^. This is not surprising, because the affinity of PEPD^G278D^ towards EGFR (Kd = 17.7 nM) [20] is much lower than that of high-affinity EGFR ligands. Importantly, aderbasib blocks the release of HB-EGF from tumor cells and allows PEPD^G278D^ to exert strong antitumor activity in tumors overexpressing HB-EGF. Because aderbasib inhibits both ADAM10 and ADAM17 which are responsible for shedding of all seven EGFR ligands, aderbasib may sensitize tumors overexpressing other high-affinity EGFR ligands to PEPD^G278D^. Despite strong inhibition of HB-EGF shedding by tumor cells, aderbasib as a single agent is inactive in our study. Aderbasib was previously evaluated in combination with trastuzumab, an HER2-directed MAB, in patients with HER2-positive breast cancer, showing inhibition of shedding of HER2 extracellular domain, and was well tolerated [46]. Aderbasib is currently evaluated in a phase 1 trial in children with recurrent and progressive high-grade gliomas (NCT04295759). 5-FU, which is commonly used for CRC treatment and has a mechanism of action distinct from that of PEPD^G278D^ and aderbasib, further enhances tumor inhibition when combined with these agents. It will be interesting to investigate whether other chemotherapeutic agents used in CRC treatment also enhance tumor inhibition when combined with the PEPD^G278D^-based treatment.

KRAS, BRAF and PIK3CA mutations occur in 32–46%, 4–16% and 13% of CRC tumors, respectively [9, 10, 47]. Patients whose tumors carry activating mutations of these genes have poor outcomes. Much effort has been devoted to developing agents targeting these oncogenic drivers, but these agents have not produced anticipated substantial clinical benefits. KRAS^G12C^ inhibitor sotorasib, which was recently approved for non-small-cell lung cancer, achieves objective response rate of only 7.1% in CRC patients whose tumors harbor this mutation [13]. Pharmacologic inhibition of BRAF^V600E^ also shows poor efficacy in CRC, with only 5.3% of patients showing partial response [14], attributed in part to feedback activation of EGFR [48]. The overall response rate to PI3K inhibitor alpelisib in CRC patients is only 5.7% [15]. The dismal outcomes of targeting mutated KRAS, BRAF and PI3K do not support the argument that mutations in these genes are critical drivers of CRC drug resistance. Our present study also shows that activating mutations of KRAS, BRAF and PIK3CA do not sustain CRC cells if EGFR and/or HER2 are depleted by PEPD^G278D^. KRAS mutations in CRC typically occur in codons 12 and 13, and PEPD^G278D^ is active in CRC cells carrying either type of KRAS mutation. BRAF^V600E^ mutation is the predominant BRAF mutation in CRC [49], and PEPD^G278D^ is active in cells carrying this mutation. PIK3CA mutation in either its catalytic domain (H1047R) or C2 domain (P449T) does not confer resistance to PEPD^G278D^ either. Most PIK3CA mutations in CRC occur in its helical domain [50]. Although we have not evaluated the effect of PIK3CA helical domain mutation on PEPD^G278D^ activity in CRC cells, we previously showed that PIK3CA helical domain mutation does not confer resistance to PEPD^G278D^ in HER2-positive breast cancer cells [21].

## Conclusions

EGFR remains the critical therapeutic target in CRC cells and tumors that are resistant to cetuximab and panitumumab and carry activating mutations of KRAS, BRAF and PIK3CA. Inducing EGFR degradation, thereby abolishing both its tyrosine kinase function and kinase-independent scaffolding function (heterodimerization with other RTKs), is critical for inhibition of CRC tumors. Inducing HER2 degradation leads to downregulation of HRAS and NRAS. PEPD^G278D^ is highly promising for overcoming CRC resistance to anti-EGFR therapies by depleting both EGFR and HER2. Combination of PEPD^G278D^ with aderbasib allows target engagement by PEPD^G278D^ in tumors overexpressing high-affinity EGFR ligands, and adding 5-FU to the combination further enhances therapeutic outcome.

## Supplementary Information

**Additional file 1.**

## Data Availability

Further information and requests for resources and reagents should be directed to and will be fulfilled by the Lead Contact, YZ (yuesheng.zhang@vcuhealth.org, or yuesheng.zhang@roswellpark.org).

## Abbreviations

ADAM10: a disintegrin and metalloproteinase domain-containing protein 10
AREG: amphiregulin
DMEM: Dulbecco’s Modified Eagle Medium
EP: enoxaparin
FBS: fetal bovine serum
HB-EGF: heparin-binding EGF-like growth factor
CRC: colorectal cancer
DMSO: dimethyl sulfoxide
EGF: epidermal growth factor
EGFR: epidermal growth factor receptor
5-FU: fluorouracil
HRP: horseradish peroxidase
IGF1R: insulin-like growth factor 1 receptor
IP: immunoprecipitation
LAMP1: lysosomal-associated membrane protein 1
MAB: monoclonal antibody
MTT: methylthiazolyldiphenyl-tetrazolium bromide
PEPD: peptidase D
PBS: phosphate-buffered saline
PDX: patient-derived xenograft
PMSF: phenylmethanesulfonyl fluoride
RT: room temperature
RTK: receptor tyrosine kinase
SDS: sodium dodecyl sulfate
TKI: tyrosine kinase inhibitor
WT: wild type

## References

[1] Spano JP, Lagorce C, Atlan D, Milano G, Domont J, Benamouzig R (2005). Impact of EGFR expression on colorectal cancer patient prognosis and survival. Ann Oncol.

[2] Shia J, Klimstra DS, Li AR, Qin J, Saltz L, Teruya-Feldstein J (2005). Epidermal growth factor receptor expression and gene amplification in colorectal carcinoma: an immunohistochemical and chromogenic in situ hybridization study. Mod Pathol.

[3] Chan DLH, Segelov E, Wong RS, Smith A, Herbertson RA, Li BT (2017). Epidermal growth factor receptor (EGFR) inhibitors for metastatic colorectal cancer. Cochrane Database Syst Rev.

[4] Cunningham D, Humblet Y, Siena S, Khayat D, Bleiberg H, Santoro A (2004). Cetuximab monotherapy and cetuximab plus irinotecan in irinotecan-refractory metastatic colorectal cancer. N Engl J Med.

[5] Van Cutsem E, Peeters M, Siena S, Humblet Y, Hendlisz A, Neyns B (2007). Open-label phase III trial of panitumumab plus best supportive care compared with best supportive care alone in patients with chemotherapy-refractory metastatic colorectal cancer. J Clin Oncol.

[6] Price TJ, Peeters M, Kim TW, Li J, Cascinu S, Ruff P (2014). Panitumumab versus cetuximab in patients with chemotherapy-refractory wild-type KRAS exon 2 metastatic colorectal cancer (ASPECCT): a randomised, multicentre, open-label, non-inferiority phase 3 study. Lancet Oncol..

[7] Bardelli A, Siena S (2010). Molecular mechanisms of resistance to cetuximab and panitumumab in colorectal cancer. J Clin Oncol.

[8] Van Emburgh BO, Sartore-Bianchi A, Di Nicolantonio F, Siena S, Bardelli A (2014). Acquired resistance to EGFR-targeted therapies in colorectal cancer. Mol Oncol.

[9] Bylsma LC, Gillezeau C, Garawin TA, Kelsh MA, Fryzek JP, Sangare L (2020). Prevalence of RAS and BRAF mutations in metastatic colorectal cancer patients by tumor sidedness: a systematic review and meta-analysis. Cancer Med.

[10] Misale S, Di Nicolantonio F, Sartore-Bianchi A, Siena S, Bardelli A (2014). Resistance to anti-EGFR therapy in colorectal cancer: from heterogeneity to convergent evolution. Cancer Discov.

[11] Karapetis CS, Khambata-Ford S, Jonker DJ, O'Callaghan CJ, Tu D, Tebbutt NC (2008). K-ras mutations and benefit from cetuximab in advanced colorectal cancer. N Engl J Med.

[12] Amado RG, Wolf M, Peeters M, Van Cutsem E, Siena S, Freeman DJ (2008). Wild-type KRAS is required for panitumumab efficacy in patients with metastatic colorectal cancer. J Clin Oncol.

[13] Hong DS, Fakih MG, Strickler JH, Desai J, Durm GA, Shapiro GI (2020). KRAS(G12C) inhibition with sotorasib in advanced solid tumors. N Engl J Med.

[14] Kopetz S, Desai J, Chan E, Hecht JR, O'Dwyer PJ, Lee RJ, et al. PLX4032 in metastatic colon cancer patients with mutant BRAF tumors. J Clin Oncol. 2010;28(15_suppl):3534.

[15] Juric D, Rodon J, Tabernero J, Janku F, Burris HA, Schellens JHM (2018). Phosphatidylinositol 3-kinase alpha-selective inhibition with alpelisib (BYL719) in PIK3CA-altered solid tumors: results from the first-in-human study. J Clin Oncol.

[16] Ramanathan RK, Hwang JJ, Zamboni WC, Sinicrope FA, Safran H, Wong MK (2004). Low overexpression of HER-2/neu in advanced colorectal cancer limits the usefulness of trastuzumab (Herceptin) and irinotecan as therapy. A phase II trial. Cancer Investig.

[17] Delord JP, Argiles G, Fayette J, Wirth L, Kasper S, Siena S (2020). A phase 1b study of the MET inhibitor capmatinib combined with cetuximab in patients with MET-positive colorectal cancer who had progressed following anti-EGFR monoclonal antibody treatment. Investig New Drugs.

[18] Rimassa L, Bozzarelli S, Pietrantonio F, Cordio S, Lonardi S, Toppo L (2019). Phase II study of tivantinib and cetuximab in patients with KRAS wild-type metastatic colorectal cancer with acquired resistance to EGFR inhibitors and emergence of MET overexpression: lesson learned for future trials with EGFR/MET dual inhibition. Clin Colorectal Cancer.

[19] Yang L, Li Y, Zhang Y (2014). Identification of prolidase as a high affinity ligand of the ErbB2 receptor and its regulation of ErbB2 signaling and cell growth. Cell Death Dis.

[20] Yang L, Li Y, Bhattacharya A, Zhang Y (2016). Dual inhibition of ErbB1 and ErbB2 in cancer by recombinant human prolidase mutant hPEPD-G278D. Oncotarget..

[21] Yang L, Li Y, Bhattacharya A, Zhang Y. A recombinant human protein targeting HER2 overcomes drug resistance in HER2-positive breast cancer. Sci Transl Med. 2019;11:eaav1620. 10.1126/scitranslmed.aav1620 PMC6409101 30674653

[22] Yang L, Li Y, Bhattacharya A, Zhang Y (2015). Inhibition of ERBB2-overexpressing tumors by recombinant human prolidase and its enzymatically inactive mutant. EBioMedicine..

[23] Witters L, Scherle P, Friedman S, Fridman J, Caulder E, Newton R (2008). Synergistic inhibition with a dual epidermal growth factor receptor/HER-2/neu tyrosine kinase inhibitor and a disintegrin and metalloprotease inhibitor. Cancer Res.

[24] Sahin U, Weskamp G, Kelly K, Zhou HM, Higashiyama S, Peschon J (2004). Distinct roles for ADAM10 and ADAM17 in ectodomain shedding of six EGFR ligands. J Cell Biol.

[25] Sahin U, Blobel CP (2007). Ectodomain shedding of the EGF-receptor ligand epigen is mediated by ADAM17. FEBS Lett.

[26] Yang L, Li Y, Ding Y, Choi KS, Kazim AL, Zhang Y (2013). Prolidase directly binds and activates epidermal growth factor receptor and stimulates downstream signaling. J Biol Chem.

[27] Troiani T, Napolitano S, Vitagliano D, Morgillo F, Capasso A, Sforza V (2014). Primary and acquired resistance of colorectal cancer cells to anti-EGFR antibodies converge on MEK/ERK pathway activation and can be overcome by combined MEK/EGFR inhibition. Clin Cancer Res.

[28] Kennedy SP, Hastings JF, Han JZ, Croucher DR (2016). The under-appreciated promiscuity of the epidermal growth factor receptor family. Front Cell Dev Biol.

[29] Kennedy SP, Han JZR, Portman N, Nobis M, Hastings JF, Murphy KJ (2019). Targeting promiscuous heterodimerization overcomes innate resistance to ERBB2 dimerization inhibitors in breast cancer. Breast Cancer Res.

[30] Young A, Lou D, McCormick F (2013). Oncogenic and wild-type Ras play divergent roles in the regulation of mitogen-activated protein kinase signaling. Cancer Discov..

[31] Zhao L, Vogt PK (2008). Helical domain and kinase domain mutations in p110alpha of phosphatidylinositol 3-kinase induce gain of function by different mechanisms. Proc Natl Acad Sci U S A.

[32] Hon WC, Berndt A, Williams RL (2012). Regulation of lipid binding underlies the activation mechanism of class IA PI3-kinases. Oncogene..

[33] Arena S, Bellosillo B, Siravegna G, Martinez A, Canadas I, Lazzari L (2015). Emergence of multiple EGFR extracellular mutations during cetuximab treatment in colorectal cancer. Clin Cancer Res.

[34] Price T, Ang A, Boedigheimer M, Kim TW, Li J, Cascinu S (2020). Frequency of S492R mutations in the epidermal growth factor receptor: analysis of plasma DNA from patients with metastatic colorectal cancer treated with panitumumab or cetuximab monotherapy. Cancer Biol Ther.

[35] Ogiso H, Ishitani R, Nureki O, Fukai S, Yamanaka M, Kim JH (2002). Crystal structure of the complex of human epidermal growth factor and receptor extracellular domains. Cell..

[36] Garrett TP, McKern NM, Lou M, Elleman TC, Adams TE, Lovrecz GO (2002). Crystal structure of a truncated epidermal growth factor receptor extracellular domain bound to transforming growth factor alpha. Cell..

[37] Li S, Schmitz KR, Jeffrey PD, Wiltzius JJ, Kussie P, Ferguson KM (2005). Structural basis for inhibition of the epidermal growth factor receptor by cetuximab. Cancer Cell.

[38] Yang L, Li Y, Bhattacharya A, Zhang Y (2016). A plasma proteolysis pathway comprising blood coagulation proteases. Oncotarget..

[39] Sanders JM, Wampole ME, Thakur ML, Wickstrom E (2013). Molecular determinants of epidermal growth factor binding: a molecular dynamics study. PLoS One.

[40] Normanno N, Tejpar S, Morgillo F, De Luca A, Van Cutsem E, Ciardiello F (2009). Implications for KRAS status and EGFR-targeted therapies in metastatic CRC. Nat Rev Clin Oncol.

[41] De Roock W, Claes B, Bernasconi D, De Schutter J, Biesmans B, Fountzilas G (2010). Effects of KRAS, BRAF, NRAS, and PIK3CA mutations on the efficacy of cetuximab plus chemotherapy in chemotherapy-refractory metastatic colorectal cancer: a retrospective consortium analysis. Lancet Oncol.

[42] Okada Y, Kimura T, Nakagawa T, Okamoto K, Fukuya A, Goji T (2017). EGFR downregulation after anti-EGFR therapy predicts the antitumor effect in colorectal cancer. Mol Cancer Res.

[43] Hecht JR, Mitchell E, Neubauer MA, Burris HA, Swanson P, Lopez T (2010). Lack of correlation between epidermal growth factor receptor status and response to Panitumumab monotherapy in metastatic colorectal cancer. Clin Cancer Res.

[44] Wheeler DL, Huang S, Kruser TJ, Nechrebecki MM, Armstrong EA, Benavente S (2008). Mechanisms of acquired resistance to cetuximab: role of HER (ErbB) family members. Oncogene..

[45] Liao HW, Hsu JM, Xia W, Wang HL, Wang YN, Chang WC (2015). PRMT1-mediated methylation of the EGF receptor regulates signaling and cetuximab response. J Clin Invest.

[46] Friedman S, Levy R, Garrett W, Doval D, Bondarde S, Sahoo T, et al. Clinical benefit of INCB7839, a potent and selective inhibitor of ADAM10 and ADAM17, in combination with trastuzumab in metastatic HER2 positive breast cancer patients. Cancer Res. 2009;69(24 suppl), abstract nr 5056.

[47] Jin J, Shi Y, Zhang S, Yang S (2020). PIK3CA mutation and clinicopathological features of colorectal cancer: a systematic review and Meta-analysis. Acta Oncol.

[48] Prahallad A, Sun C, Huang S, Di Nicolantonio F, Salazar R, Zecchin D (2012). Unresponsiveness of colon cancer to BRAF(V600E) inhibition through feedback activation of EGFR. Nature..

[49] Jones JC, Renfro LA, Al-Shamsi HO, Schrock AB, Rankin A, Zhang BY (2017). ^Non-V600^BRAF mutations define a clinically distinct molecular subtype of metastatic colorectal cancer. J Clin Oncol.

[50] Wang Q, Shi YL, Zhou K, Wang LL, Yan ZX, Liu YL (2018). PIK3CA mutations confer resistance to first-line chemotherapy in colorectal cancer. Cell Death Dis.

