# Supplementary material for: Depleting receptor tyrosine kinases EGFR and HER2 overcomes resistance to EGFR inhibitors in colorectal cancer
Source: J Exp Clin Cancer Res. 2022 Jun 2;41:184. doi: 10.1186/s13046-022-02389-z (PMC9161494; doi:10.1186/s13046-022-02389-z)
Supplement: Supplementary file 1 — Additional file 1. [file 13046_2022_2389_MOESM1_ESM.pdf]

Supplementary Figures

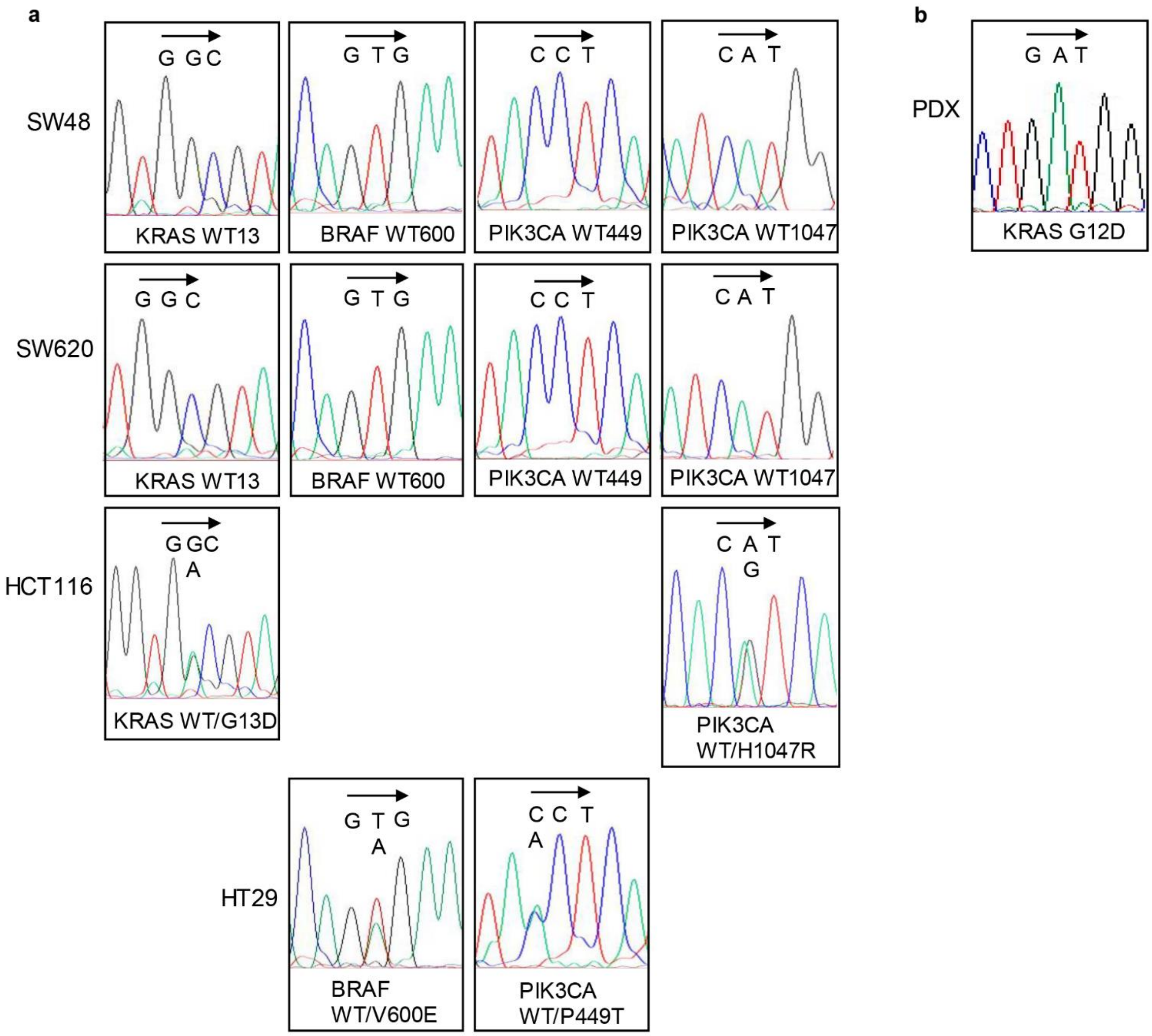

Fig. S1 KRAS, BRAF and/or PIK3CA are mutated in two CRC cell lines and PDX14650. **a** Gene mutation analysis in cell lines, including KRAS, BRAF, and PIK3CA. **b** Analysis of KRAS mutation in PDX14650.

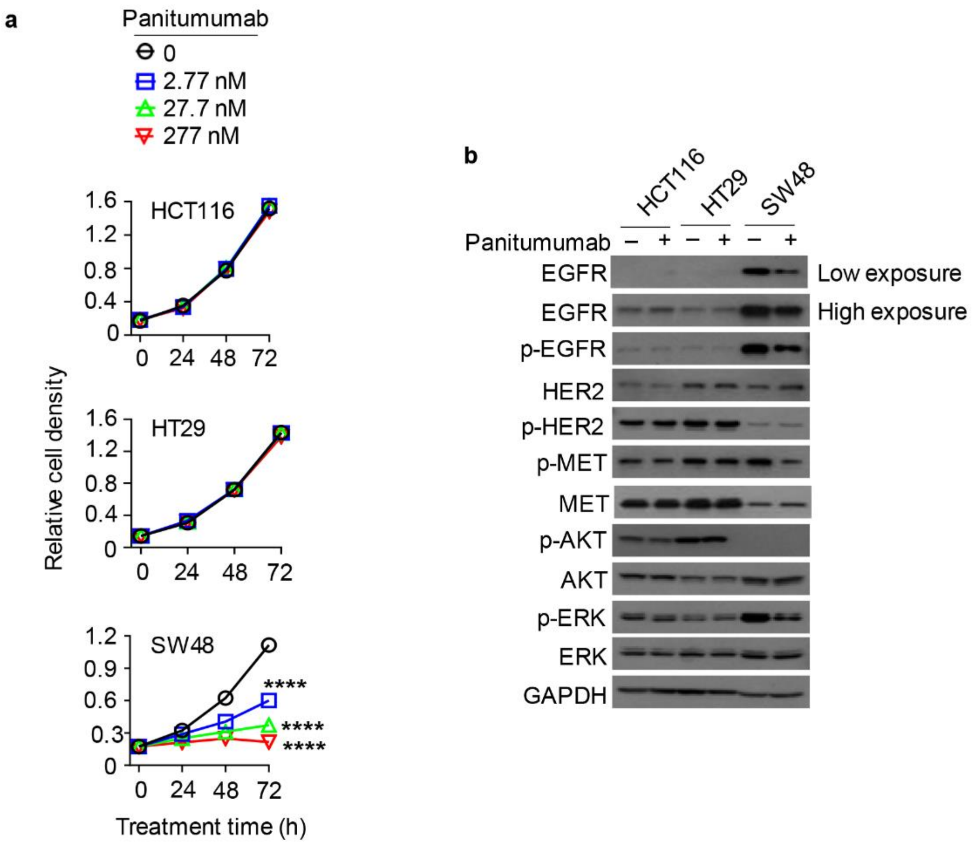

Fig. S2 The growth-inhibitory activity of panitumumab in CRC cell lines correlates with EGFR downregulation. **a** Effects of panitumumab on cell growth measured by MTT assay. Each value is mean ± SD (n = 3). ****P < 0.0001 by one-way ANOVA, followed by Tukey test for comparison with the control. **b** Western blotting of whole cell lysates after treatment of the cells with vehicle or panitumumab (277 nM) for 48 h.

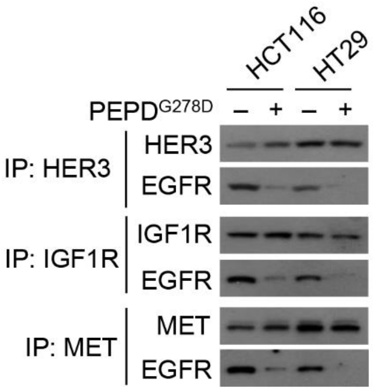

Fig. S3 PEPD^{G278D} disrupts EGFR-HER3, EGFR-IGF1R and EGFR-MET heterodimers. Western blotting of anti-HER3 IP, anti-IGF1R IP or anti-MET IP of whole cell lysates from cells treated with PEPD^{G278D} (25 nM) for 48 h.

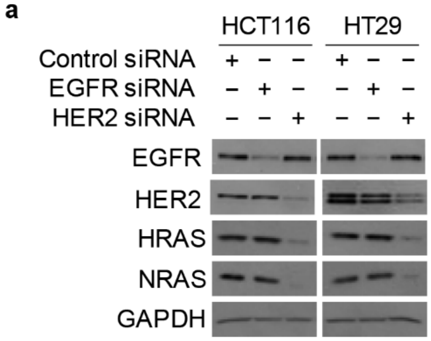

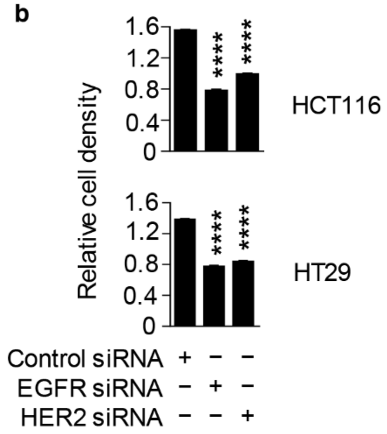

Fig. S4 CRC cells resistant to EGFR MABs require EGFR and HER2. **a** Western blotting of whole cell lysates after treatment of cells with EGFR siRNA or HER2 siRNA for 48 h. **b** Effects of knockdown of EGFR or HER2 by siRNA on cell growth measured by MTT assay. Each value is mean ± SD (n = 3). ****P<0.0001 by two-tailed unpaired t test.

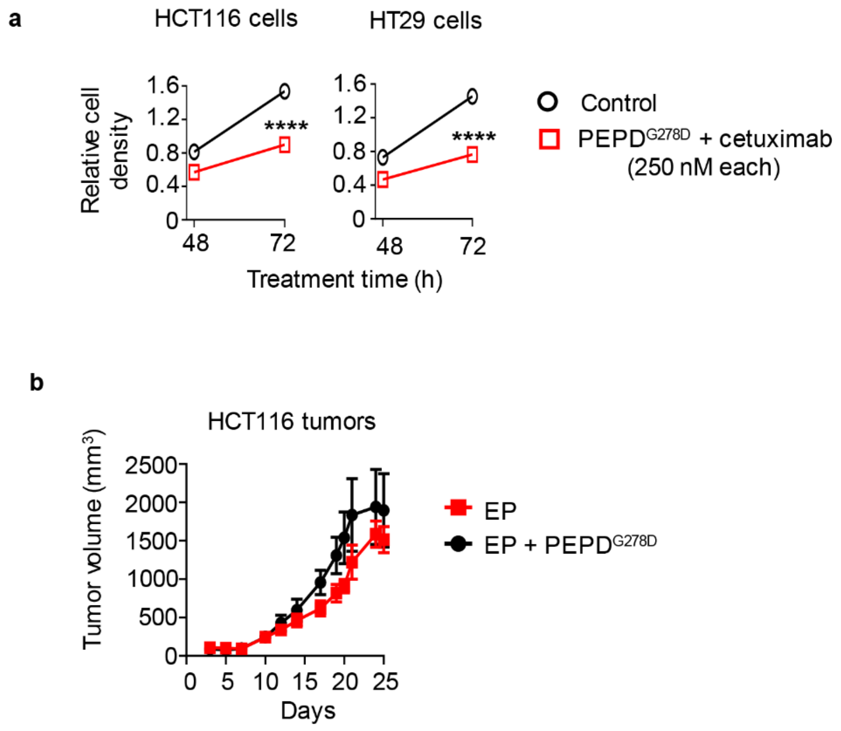

Fig. S5 Cetuximab attenuates cell growth inhibition by PEPD^{G278D} in vitro, and EP plus high dose of PEPD^{G278D} do not inhibit tumor growth in vivo. **a** Effect of combining PEPD^{G278D} with cetuximab on cell growth measured by MTT assay. Each value is mean ± SD (n = 3). ****P<0.0001 by two-tailed unpaired t test. **b** Mice bearing subcutaneous HCT116 tumors were randomized to EP, or EP plus PEPD^{G278D}. EP was administered to mice at 0.5 mg/kg per dose daily by ip (days 3-24). PEPD^{G278D} was administered to mice at 8 mg/kg per dose thrice weekly by ip (days 10-24). Each value is mean ± SEM (n = 6).

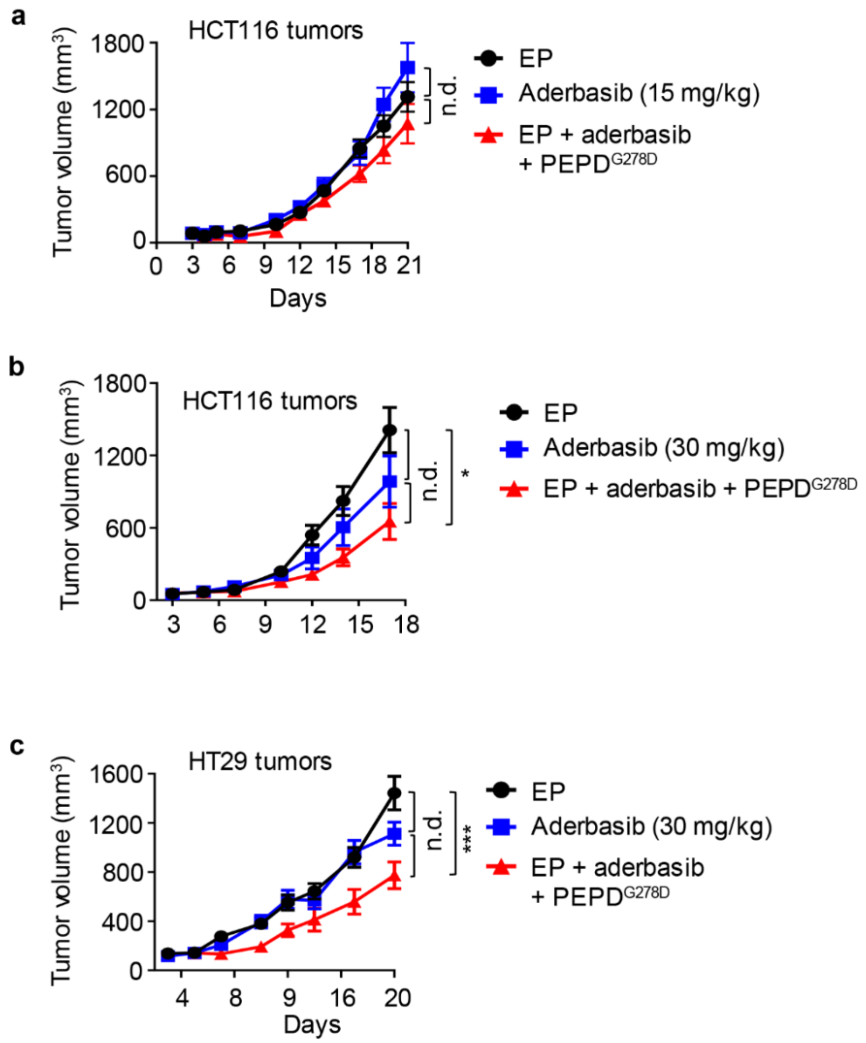

Fig. S6 Aderbasib restores the antitumor activity of PEPD^{G278D} in a dose-dependent manner. **a-c** Mice bearing subcutaneous tumors were randomized to EP (n = 12-16), aderbasib (n = 12), or EP plus aderbasib plus PEPD^{G278D} (n = 12). EP was administered to mice daily by ip at 0.5 mg/kg per dose (HCT116 tumors in **a**: days 3-20; HCT116 tumors in **b**: days 2-16; HT29 tumors in **c**: days 2-19). Aderbasib was administered to mice daily by gavage at 15 or 30 mg/kg per dose (HCT116 tumors in **a**: days 4-20; HCT116 tumors in **b**: days 4-16; HT29 tumors in **c**: days 4-19). PEPD^{G278D} was administered to mice thrice weekly by ip at 4 mg/kg per dose (HCT116 tumors in **a**: days 5-20; HCT116 tumors in **b**: days 5-16; HT29 tumors in **c**: days 5-19). Each value is mean ± SEM. *P < 0.01, ***P < 0.001, n.d., not different, by one-way ANOVA, followed by Tukey test.

a

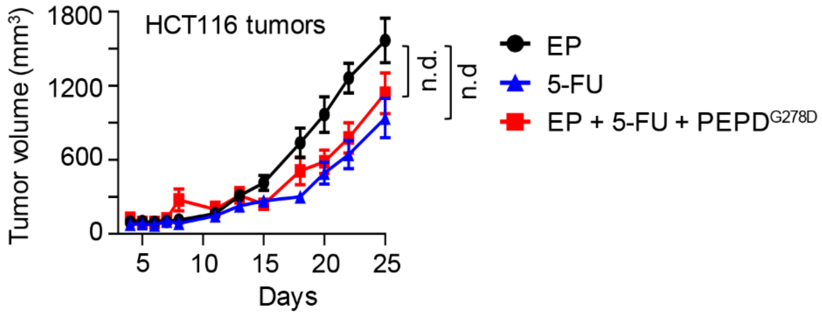

b

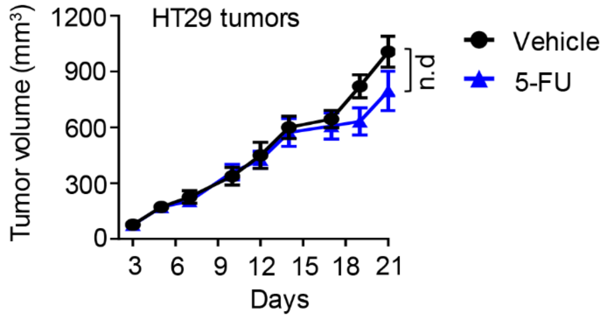

Fig. S7 5-FU alone or in combination with EP and PEPD^{G278D} without aderbasib does not inhibit tumor growth. **a** Mice bearing subcutaneous HCT116 tumors were randomized to EP (n = 6), 5-FU (n = 6), or EP plus 5-FU plus PEPD^{G278D} (n = 6). EP was administered to mice daily by ip at 0.5 mg/kg per dose (days 4-24). 5-FU was administered to mice by ip every 3-4 days at 35 mg/kg per dose (days 7-24). PEPD^{G278D} was administered to mice by ip thrice weekly at 4 mg/kg per dose (days 6-24). **b** Mice bearing subcutaneous HT29 tumors were randomized to vehicle (n = 14) or 5-FU (n =16). Vehicle and 5-FU at 35 mg/kg per dose were administered to mice by ip every 4 days (days 6 to 18). Each value is mean ± SEM. n.d., not different.

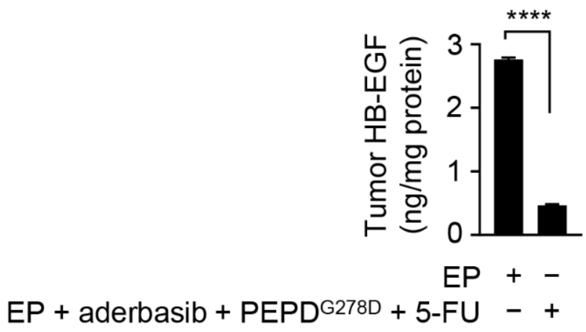

Fig. S8 HB-EGF levels in PDX14650 tumors. Tumor samples were from the experiment described in Fig. 6C. Tumor levels of soluble HB-EGF (three tumors per group) were measured by ELISA. Each value is mean ± SD. ****P<0.0001 by t-test.

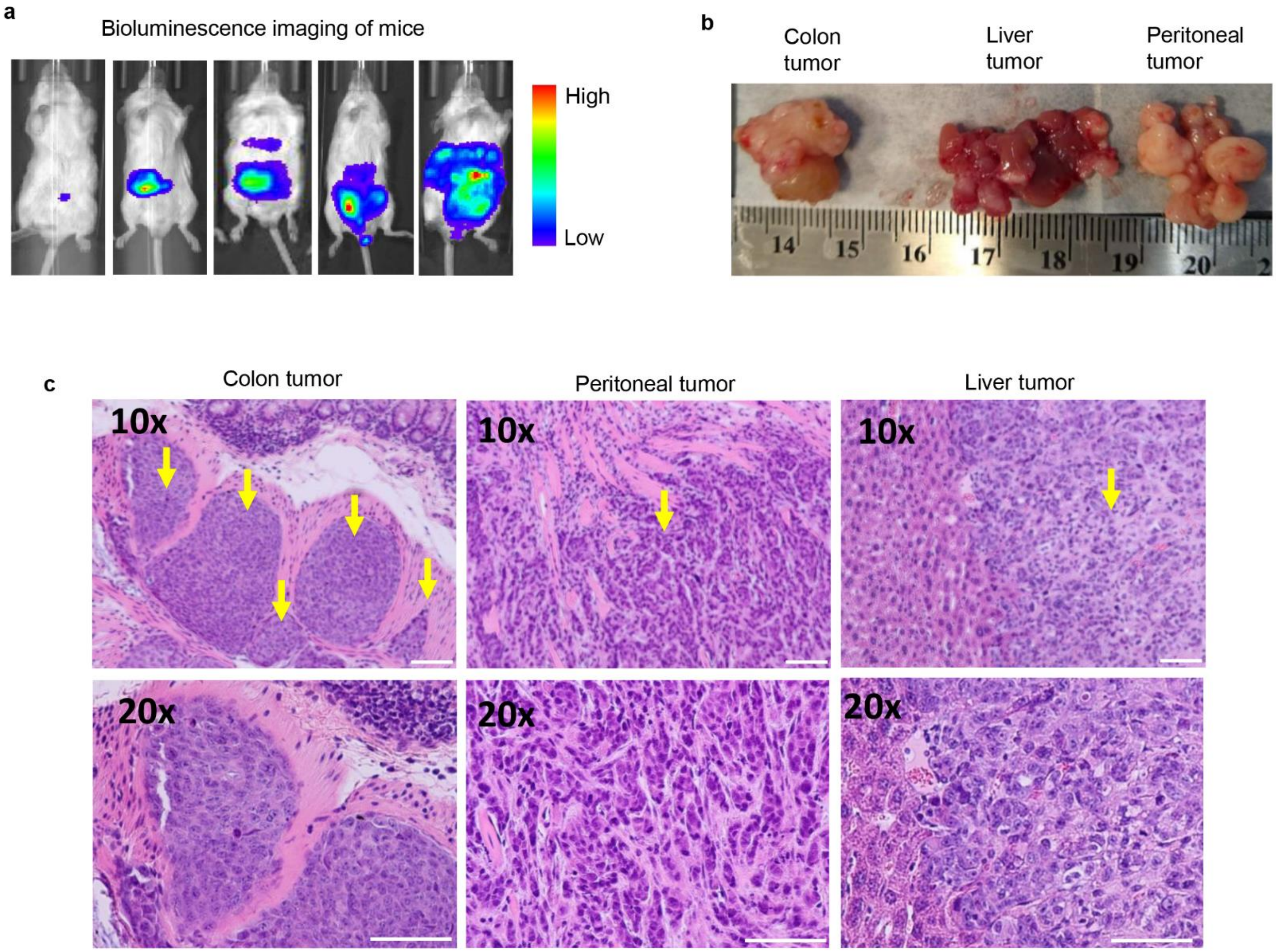

Fig. S9 Supporting data for the experiment shown in Fig. 7. **a** Whole-body bioluminescence imaging of representative mice on day 56. **b** Representative colon, liver and peritoneal tumors. **c** H & E staining of representative tumors. Scale bar: 100 μm.
